# Effect of supplementation with *L*-Citrulline on rumen microbiota structure, plasma metabolites, reproductive hormones, and antioxidant capacity of Hu ewes

**DOI:** 10.3389/fmicb.2025.1606437

**Published:** 2025-06-25

**Authors:** Renping Liu, Peiyao Li, Chen Fan, Shanshan Wang, Zhiqiang Liu, Guodong Zhao, Kailun Yang

**Affiliations:** ^1^College of Animal Science, Xinjiang Agricultural University, Urumqi, China; ^2^Xinjiang Shangpin Meiyang Technology Co., Ltd., Changji, China

**Keywords:** *L*-Citrulline, rumen microbiota, untargeted metabolomics, volatile fatty acids, reproductive hormones, antioxidant capacity

## Abstract

*L-*Citrulline (*L-*Cit), a non-essential amino acid, is characterized by its unique extrahepatic metabolism, which significantly enhances the bioavailability of arginine metabolism in tissues. This study investigated the impact of *L*-Cit supplementation on ruminal microbiota composition, plasma metabolites, reproductive hormones, and antioxidant capacity in Hu ewes. Sixty non-pregnant Hu ewes, similar in age and parity, with an average body weight of 47 ± 5.05 kg, were randomly assigned to either a Control group or Experimental group. The Control group received a basal diet, while the Experimental group was supplemented with 10 g/d of *L*-Cit in addition to the basal diet for 65 days. Compared to the Control group, the Experimental group exhibited a significantly higher estrus rate. Plasma estradiol (E_2_) levels were significantly reduced (*p* < 0.01), while luteinizing hormone (LH) and follicle-stimulating hormone (FSH) concentrations showed significant increases (*p* < 0.05). Testosterone (T) content was also significantly elevated (*p* < 0.01). Plasma levels of superoxide dismutase (SOD), malondialdehyde (MDA), glutathione peroxidase (GSH-PX), catalase (CAT), and total antioxidant capacity (T-AOC) were significantly higher in the experimental group, with highly significant differences (*p* < 0.01). The 16S rRNA sequencing analysis revealed that at the phylum level, the relative abundance of Bacteroidetes was decreased, while that of Firmicutes was increased in the experimental group. At the family level, the relative abundance of norank_o__Clostridia_UCG-014 was significantly increased. At the genus level, the relative abundance of Prevotellaceae_UCG-003 was significantly decreased. The main enriched pathways in the CON group were identified as Lipoic acid metabolism and Nicotinate and nicotinamide metabolism. The main enriched pathways in the experimental group were identified as Prion diseases, Chlorocyclohexane and chlorobenzene degradation, Chloroalkane and chloroalkene degradation, Biofilm formation-*Escherichia coli*, and Phosphotransferase system (PTS). LC–MS analysis indicated significant upregulation of pathways such as drug metabolism by other enzymes, folate biosynthesis, and valine, leucine, and isoleucine biosynthesis, whereas oxidative phosphorylation and propanoate metabolism were significantly downregulated. These results demonstrate that *L*-Cit supplementation in the diet modulates the ruminal microbiota of Hu ewes, optimizing volatile fatty acid (VFA) proportions, enhancing carbohydrate metabolism, improving xenobiotic degradation capacity, stimulating the synthesis and release of reproductive hormones. Ultimately, these coordinated effects led to a synergistic increase in estrus and conception rates.

## 1 Introduction

*L*-Citrulline (*L*-Cit), a non-essential amino acid, plays a pivotal role in nitric oxide (NO) synthesis and systemic metabolism via two primary pathways: arginine metabolism and NO production, which contribute to the generation of NO and polyamines (Ma et al., 2019). It has been demonstrated in studies that plasma arginine levels are increased, NO synthesis is enhanced, and vascular relaxation and exercise performance are improved through supplementation with L-citrulline (Schwedhelm et al., 2008). In addition, the urea cycle is promoted, blood ammonia levels following exercise are reduced, and the onset of fatigue is delayed through L-citrulline supplementation (Suzuki et al., 2015). Arginine serves as a key substrate for protein synthesis and the production of nitrogen-containing metabolites, supporting numerous cellular metabolic functions (Wu et al., 2018). Studies have shown that arginine availability is largely dependent on hepatic uptake (Cynober et al., 1995; Curis et al., 2005; Schwedhelm et al., 2008). In contrast, *L*-Cit, due to its distinct extrahepatic metabolic properties, significantly enhances the bioavailability of arginine in tissues (Gilbreath et al., 2020). Our previous research demonstrated that *L*-Cit supplementation (at optimal levels of 10–15 g/d per ewe) markedly improved plasma reproductive hormone levels, antioxidant capacity, and reproductive performance in Hu sheep ewes (An, 2022; Ma et al., 2023). Similar improvements were observed in rams, including enhanced blood reproductive hormone levels and antioxidant capacity (Liu, 2021; Zhao et al., 2022b). While the effects of *L*-Cit on gut microbiota restructuring in rodents have been established (Li Xiabin et al., 2019). However, the impact of L-citrulline on the rumen microbiota of Hu sheep remains unelucidated, and the dynamic alterations in the blood metabolome induced by its ingestion have not been systematically characterized. The rumen of ruminants represents a complex microbial fermentation system, where the microbiota structure and host metabolism are intricately linked. To address this gap, the current study utilized Hu sheep ewes as a model, analyzing plasma metabolomic changes through liquid chromatography-mass spectrometry (LC–MS) and characterizing the rumen microbial community using 16S rRNA high-throughput sequencing. This research aims to elucidate the impact of *L*-Cit supplementation on host metabolic regulation and ruminal microecology, thereby providing a theoretical foundation for the application of *L*-Cit in the nutritional management of ruminants.

## 2 Materials and methods

### 2.1 Experimental materials

*L*-Cit (Purity≥99.9%) was obtained from Shandong Pingju Biotechnology Co., Ltd., and cloprostenol sodium (Prostaglandin, PG) was sourced from Ningbo Second Hormone Factory.

### 2.2 Experimental design and grouping

The experiment was conducted from September to December 2024 at Xinjiang Shangpin Meiyang Technology Co., Ltd. (longitude 87.136291, latitude 44.359568). A total of sixty multiparous Hu sheep ewes, with an average body weight of 47 ± 5.05 kg and similar age and parity, were selected for the study. The ewes were randomly assigned to either a Control group (C) or an Experimental group (T), and a 65-day supplementation trial was carried out (Table 1). Day 0 was recorded as the start of formal supplementation. To induce rapid luteolysis and ensure uniform physiological status, the experimental ewes were administered intramuscular injections of PG using a two-batch method at a dose of 1.5 mL per ewe on days −7 and −3 of the trial. The Control group (C) received only the basal diet, while the Experimental group (T) was supplemented with 10 g/d of *L*-Cit per animal in addition to the basal diet, Refeed in two batches for 5 g/per ewe each time, with the dosage being determined based on previous studies (Ma et al., 2023). *L-*Cit was fully mixed with 100 g bran and a small amount of water was added to ensure that it was fed after adsorption with bran. The control group was fed 100 g bran, and TMR diet was fed after the drug was completely eaten. Supplementation ceased on day 10 of the trial. A 7-day heat detection period was conducted from days 15 to 21, and estrus rates were calculated upon completion of the detection. Pregnancy diagnosis was performed on day 30 after mating, and pregnancy rates were recorded (Supplementary Table S1).

**Table 1:** Composition and nutrient levels of TMR diet (DM basis %).

Ingredients	Content %	Nutrient levels	Content %
Whole corn silage	47.42	Organic compound	92.12
Alfalfa hay	18.58	Crude protein	11.32
Wheat straw	19.59	Neutral detergent fiber	53.35
Concentrate supplement	14.41	Acidic detergent fiber	32.28
Total	100	Ca P	0.65 0.33

Composition of the concentrate supplement: Crude Protein ≥18%; Crude Fiber ≤15%; Crude Ash ≤15%; Calcium: 0.5–2.5%; Sodium Chloride: 0.5–2.0%; Sulfur-Containing Amino Acids ≥0.4%; Moisture ≤14%; Total Phosphorus ≥0.4%.

The above values are estimate.

### 2.3 Feeding management

Throughout the experiment, the ewes in each group were housed in separate pens, with each pen subdivided into six smaller pens containing 10 ewes each. All experimental ewes were managed under identical feeding conditions, provided with a total mixed ration (TMR) diet of consistent nutritional quality, supplied by Xinjiang Shangpin Meiyang Technology Co., Ltd. The TMR diet was administered in two daily batches at 09:00 and 16:00, the ewes were allowed free access to feed and water, with a daily dry matter intake of approximately 1.65 kg per ewe (Table 1).

### 2.4 Sample collection and measurement

#### 2.4.1 Collection of blood samples

The official supplementation period commenced on day 0 of the experiment. On days −7 and −3, two doses of PG treatment were administered. Blood samples were collected from the jugular vein of the ewes on Day 10 (under the morning fasting state and 3 h post-feeding). During blood collection, the ewe was restrained with its neck extended upward, and pressure was applied to the jugular vein until it became prominent. A blood collection needle was inserted at a 45-degree angle towards the head, and once blood return was observed, the needle was placed into a heparin sodium blood collection tube. Approximately 10 mL of blood was collected into two tubes. The collected blood was centrifuged at 3500 rpm for 15 min in a high-speed centrifuge to prepare plasma. The plasma was then aliquoted into 1.8 mL cryovials, labeled according to group, and stored at −20°C for subsequent analysis.

#### 2.4.2 Determination of plasma biochemical parameters and metabolites

Plasma levels of gonadotropin-releasing hormone (GnRH), follicle-stimulating hormone (FSH), luteinizing hormone (LH), testosterone (T), and estradiol (E_2_) were measured. Antioxidant markers, including superoxide dismutase (SOD), malondialdehyde (MDA), glutathione peroxidase (GSH-PX), catalase (CAT), nitric oxide (NO), and total antioxidant capacity (T-AOC), were also assessed. Plasma LC–MS detection followed the method outlined by Xian et al. (2022). All samples were sent to Shanghai Majorbio Bio-pharm Technology Co., Ltd. for testing.

#### 2.4.3 Rumen fluid collection

On day 11 of the experiment, 100 mL of rumen fluid was collected via the oral cavity using a negative pressure extraction device, 3 h after morning feeding. To avoid saliva contamination, the first 50 mL of rumen fluid was discarded. The pH of the rumen fluid was then measured using a portable pH meter (Supplementary Table S2). The rumen fluid was subsequently filtered through four layers of medical gauze, aliquoted, and stored at −80°C.

#### 2.4.4 Measurement of rumen fluid parameters

16S rRNA sequencing was outsourced to Shanghai Majorbio Bio-pharm Technology Co., Ltd. Universal bacterial primers (338F: 5’-ACTCCTACGGGAGGCAGCAG-3′ and 806R: 5’-GGACTACHVGGGTWTCTAAT-3′) were used to amplify the V3-V4 region of the bacterial 16S rRNA via PCR. PCR products were electrophoresed on a 2% agarose gel. PCR reaction parameters were as follows: (a) 1 × (3 min at 95°C), (b) Number of cycles × (30 s at 95°C; 30 s at the annealing temperature; 45 s at 72°C), (c) 10 min at 72°C, followed by 10°C until halted by the user. The raw data has been uploaded to NCBI: https://www.ncbi.nlm.nih.gov/sra/PRJNA1231430.

### 2.5 Data analysis

Data on estrus rate, conception rate, reproductive hormones, and antioxidant markers in plasma were initially organized using Microsoft Excel 2021. Independent samples *t*-tests for reproductive hormones and antioxidant indicators were performed using IBM SPSS Statistics 27 software. Statistical analysis and graphing were carried out using GraphPad Prism 10. Analysis of rumen fluid samples and untargeted metabolomics data was performed on the cloud platform of Shanghai Majorbio Bio-pharm Technology Co., Ltd.

## 3 Results

### 3.1 Effects of *L*-Citrulline supplementation on the rumen microbial community in Hu sheep ewes

#### 3.1.1 Alpha diversity analysis

As presented in Supplementary Supplementary Table S3, no significant differences were observed between the control and experimental groups across various indices. These results suggest that dietary supplementation with *L*-Cit did not significantly alter the diversity of the rumen microbial community. Additionally, the species coverage for all groups reached 99.9%, confirming that the sequencing depth adequately captured the true composition of the microbial community in the rumen fluid samples.

#### 3.1.2 Principal component analysis

**Figure 1:**
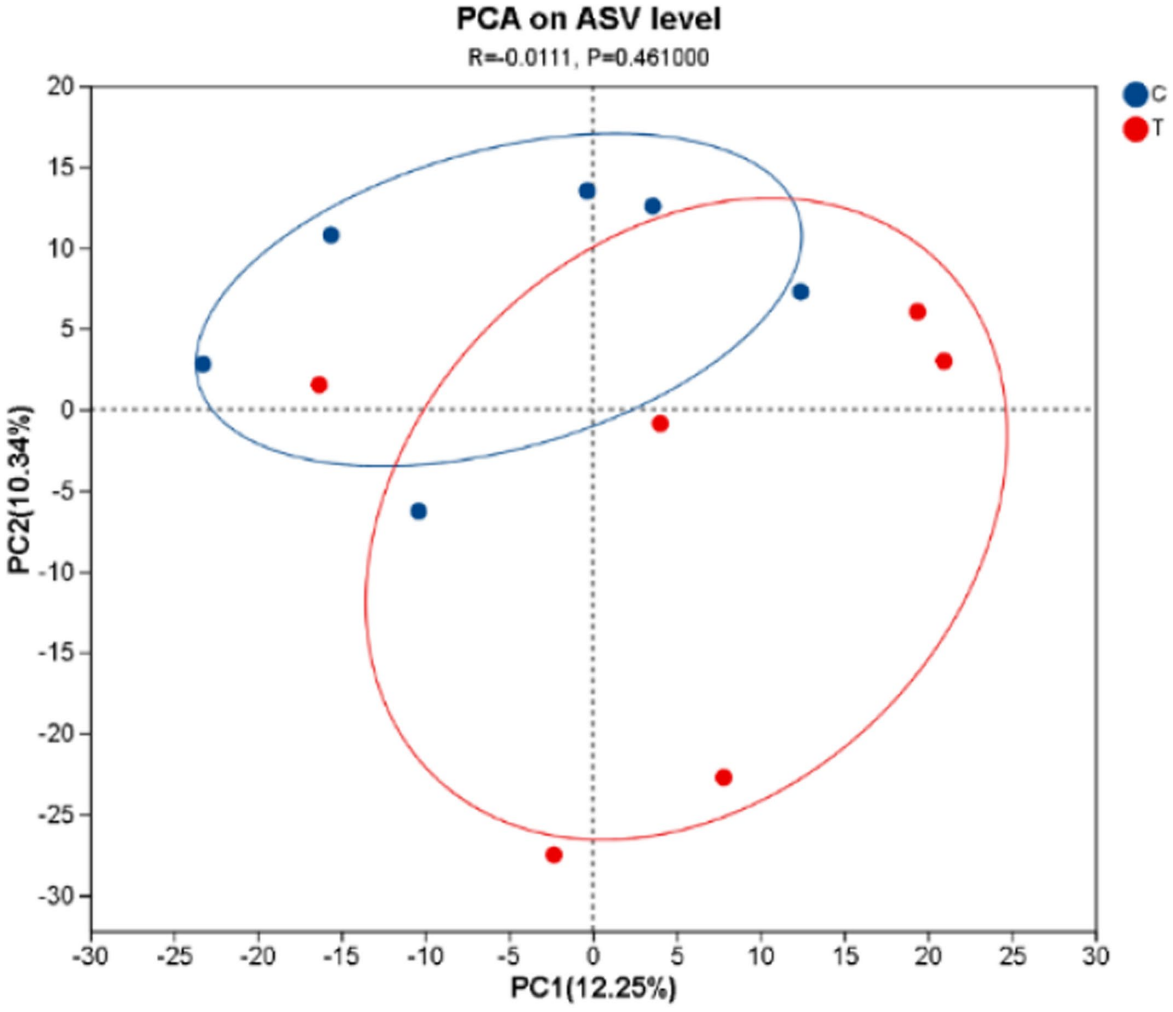
The PCA plot was generated. C denotes the control group; T represents the experimental group.

Figure 1 illustrates that the first principal component explains 12.25% of the sample variation, while the second principal component accounts for 10.34%. The CON and experimental groups form distinct species clusters, with the CON group displaying a relatively compact microbial composition, indicating smaller differences in bacterial communities. In contrast, the experimental group shows greater dispersion, suggesting a higher degree of variability in its microbial community.

#### 3.1.3 Effects of *L*-Citrulline supplementation on rumen microbiota (phylum level) in Hu sheep ewes

As shown in Supplementary Figure S1A, Bacteroidota and Firmicutes were predominantly represented among the top 10 phyla in rumen microbiota relative abundance at the phylum level, being identified as the dominant phyla in both the Control group and the Experimental group. A higher relative abundance of Bacteroidota was observed in the Control group (49.46%) compared to the Experimental group, while Firmicutes was found to be more predominant in the Experimental group (50.43%) than in the Control group.

#### 3.1.4 Effects of *L*-Citrulline supplementation on rumen microbiota (family level) in Hu sheep ewes

As shown in Supplementary Figure S1B, Prevotellaceae, Ruminococcaceae, and Lachnospiraceae were predominantly represented among the top 10 families in rumen microbiota relative abundance at the family level, being identified as the dominant families in both the Control group and the Experimental group. A higher relative abundance of Prevotellaceae was observed in the Control group (30.12%) compared to the Experimental group. Higher relative abundances of Ruminococcaceae and Lachnospiraceae were observed in the Experimental group (13.41 and 13.84%, respectively) compared to the Control group. Furthermore, a significantly higher relative abundance of norank_o__Clostridia_UCG-014 was observed in the Experimental group compared to the Control group.

#### 3.1.5 Effects of *L*-Citrulline supplementation on rumen microbiota (genus level) in Hu sheep ewes

As shown in Supplementary Figure S1C, Prevotella and Ruminococcus were predominantly represented among the top 10 genera in rumen microbiota relative abundance at the genus level, being identified as the dominant genera in both the Control group and the Experimental group. A higher relative abundance of Prevotella was observed in the Control group (23.42%) compared to the experimental group, while Ruminococcus was found to be more predominant in the Experimental group (12.61%) than in the Control group. A significantly higher relative abundance of Prevotellaceae_UCG-003 was observed in the Control group compared to the Experimental group, with a statistically significant difference.

#### 3.1.6 Comparative analysis of differential microbial species in rumen microbiota

**Figure 2:**
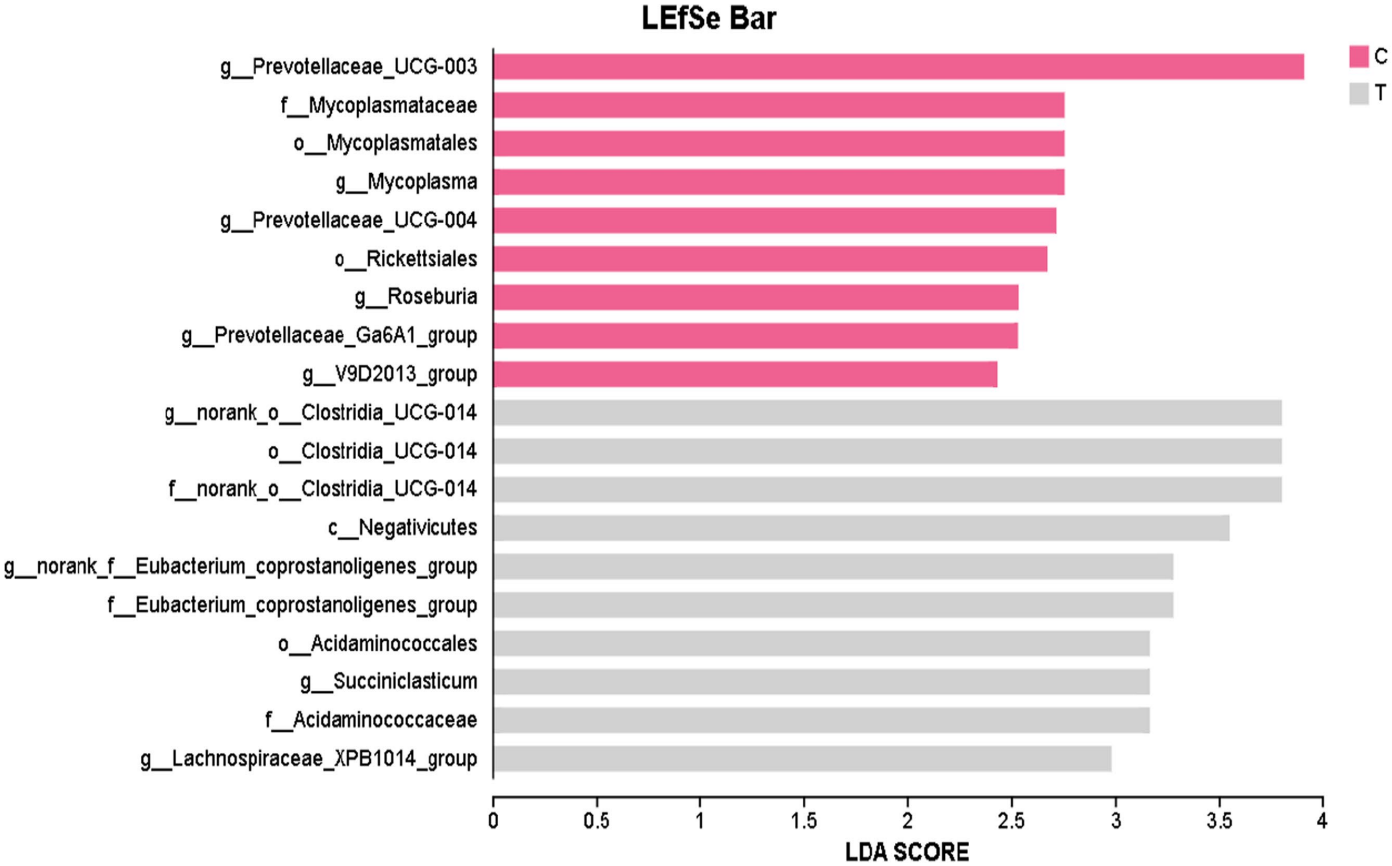
The LefSe LDA bar plot was generated to display discriminative features. The LDA discriminant bar chart statistically represents microbial taxa with significant effects across multiple groups. The LDA score, obtained through LDA analysis (linear regression analysis), indicates that the higher the LDA score, the greater the impact of species abundance on the differential effect. C denotes the control group; T represents the experimental group.

Figure 2 highlights 19 microbial taxa with significant differences between the Control group and Experimental group, including 1 class, 4 orders, 4 families, and 10 genera. In the Control group, the species Prevotellaceae_UCG-003, Mycoplasmataceae, Mycoplasmatales, Mycoplasma, Prevotellaceae_UCG-004, Rickettsiales, Roseburia, Prevotellaceae_Ga6A1_group, and V9D2013_group are prevalent. The Experimental group is dominated by the species norank_o__Clostridia_UCG-014, Clostridia_UCG-014, norank_o__Clostridia_UCG-014, Negativicutes, norank_f__Eubacterium_coprostanoligenes_group, Eubacterium_coprostanoligenes_group, Acidaminococcales, Succiniclasticum, Acidaminococcaceae, and Lachnospiraceae_XPB1014_group.

#### 3.1.7 Functional prediction

**Figure 3:**
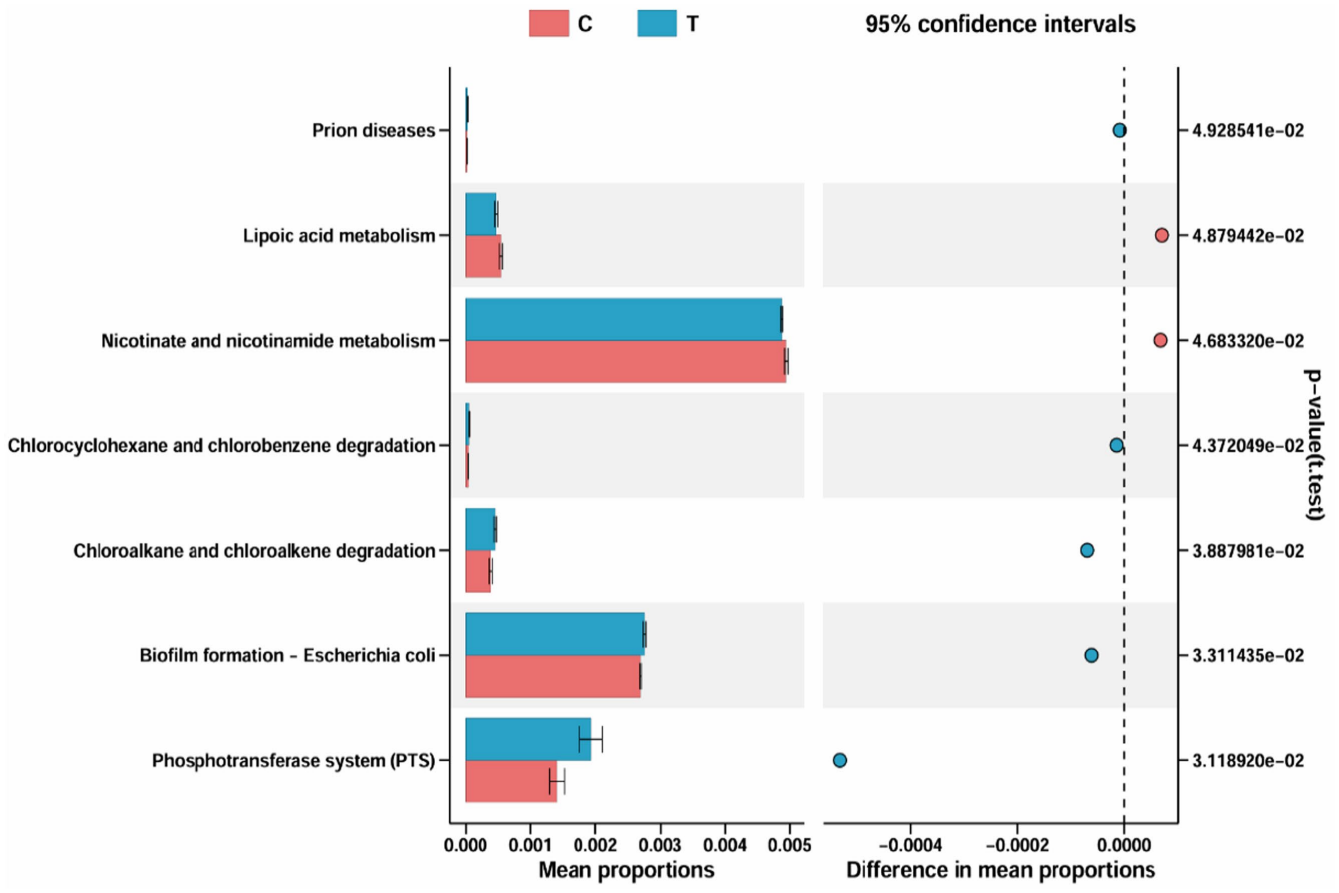
Metabolic pathway differences diagram. C denotes the control group; T represents the experimental group.

Figure 3 presents the identification of 7 differential metabolic pathways between the Control group and Experimental groups. The primary enriched pathways in the Control group are Lipoic acid metabolism and Nicotinate and nicotinamide metabolism, whereas the Experimental group shows significant enrichment in Prion diseases, Chlorocyclohexane and chlorobenzene degradation, Chloroalkane and chloroalkene degradation, *Biofilm formation – *Escherichia coli*, and Phosphotransferase system (PTS).

### 3.2 Effects of *L*-Citrulline supplementation on volatile fatty acid profiles in the rumen of Hu sheep ewes

**Figure 4:**
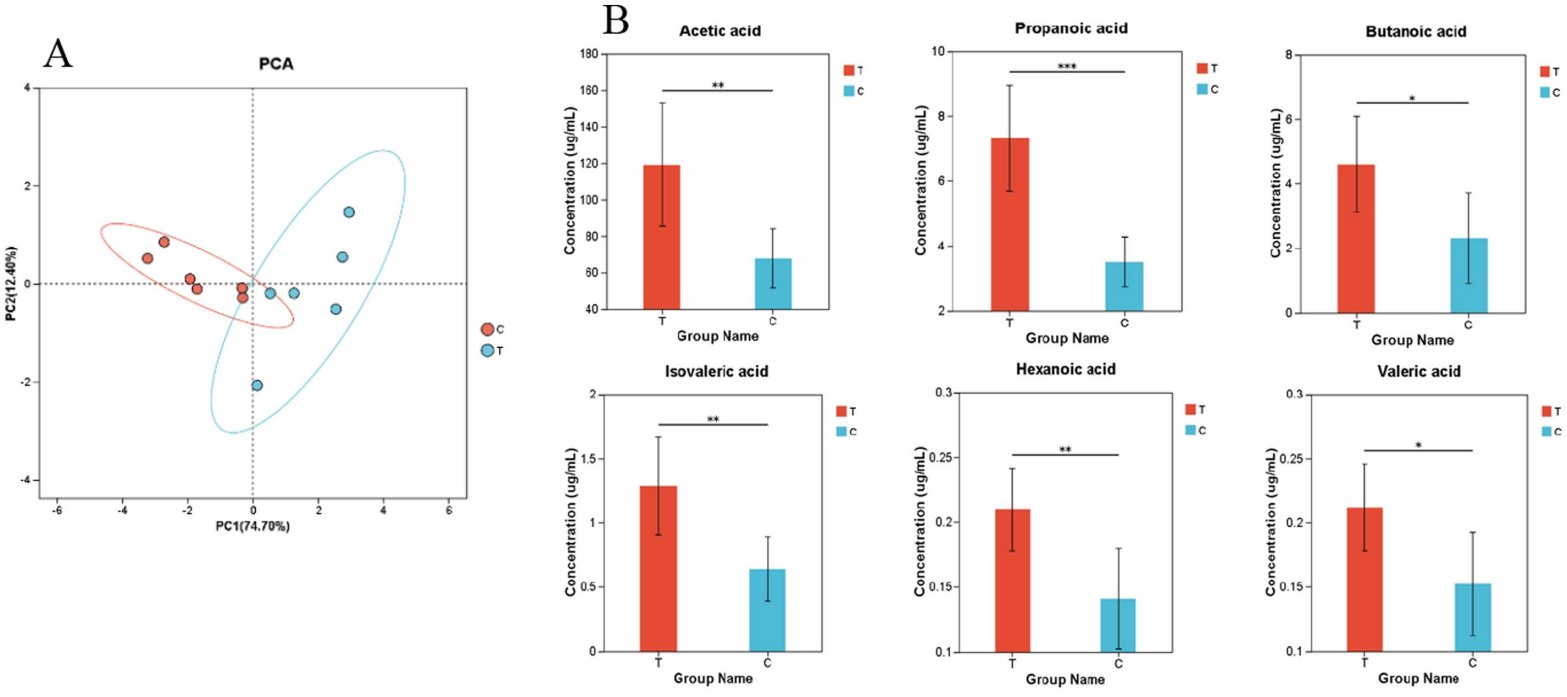
**(A)** Principal Component Analysis **(B)** Bar Chart of Relative Abundance of Volatile Fatty Acids. **(B)** The bar chart represents the relative expression abundance of metabolites in each group. The horizontal axis denotes the different groups, while the vertical axis represents the mass spectrometry intensity values (preprocessed mass spectrometry intensity). The error bars indicate the mean ± standard deviation. Significant differences are marked with asterisks (*0.01 < *p* ≤ 0.05, **0.001 < *p* ≤ 0.01, ***p ≤ 0.001). **(A,B)** C denotes the control group; T represents the experimental group.

As shown in Figure 4A, the first principal component accounts for 74.70% of the sample variation, while the second principal component accounts for 12.40%. The Experimental group exhibits a more dispersed structure and greater variability compared to the Control group.

Figure 4B demonstrates that the relative abundances of acetic acid, propanoic acid, isovaleric acid, and hexanoic acid are significantly higher in the Experimental group than in the Control group. Additionally, the relative abundances of butanoic acid and valeric acid are also significantly elevated in the Experimental group compared to the Control group.

It was found through calculations that the rumen fermentation types of both the control and experimental groups were acetate-type (Supplementary Table S4).

### 3.3 Impact of *L*-Citrulline supplementation on plasma metabolites in Hu sheep ewes

#### 3.3.1 Partial least squares discriminant analysis

**Figure 5:**
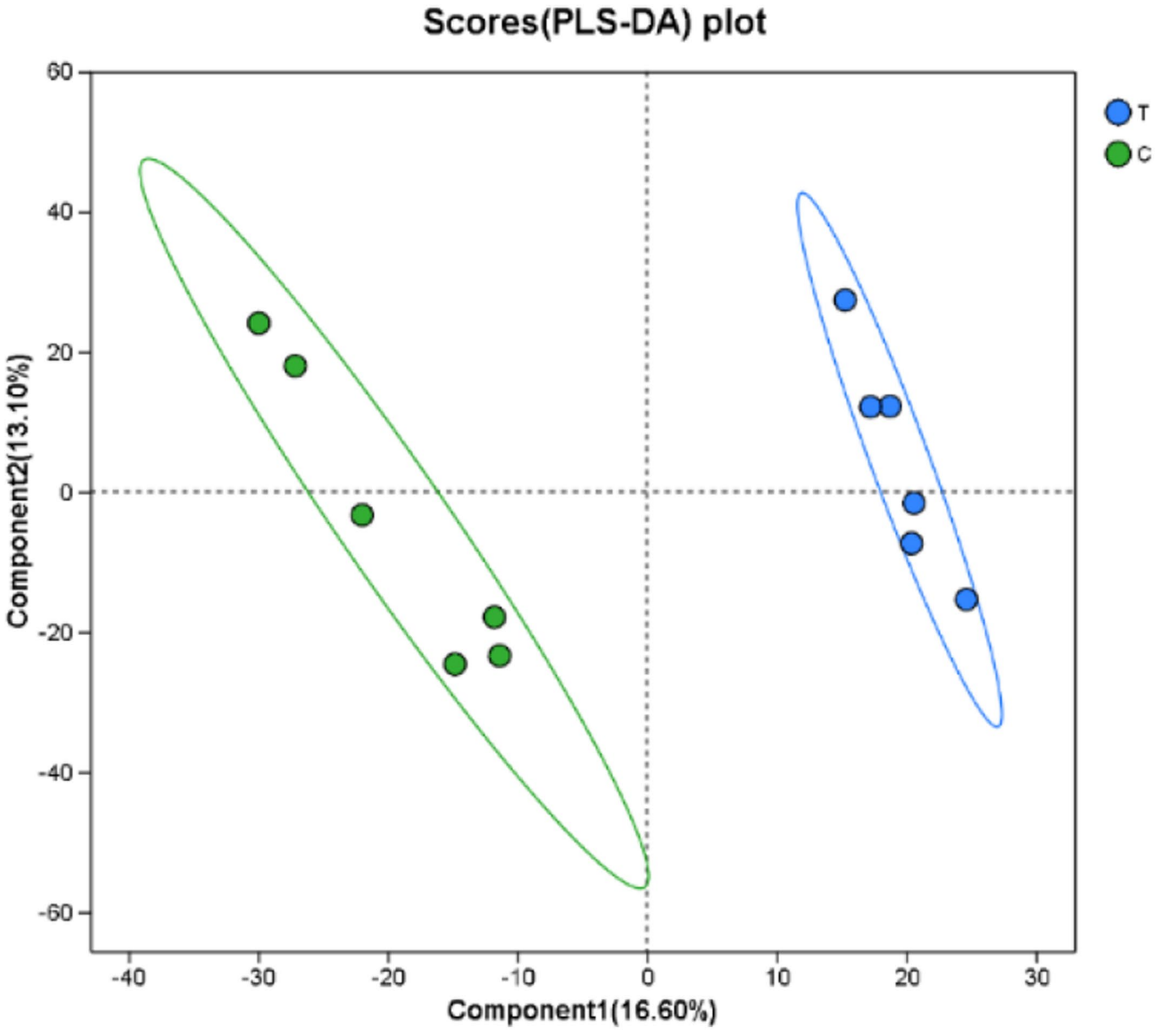
PLS-DA score chart. C denotes the control group; T represents the experimental group.

As presented in Figure 5, the PLS-DA score plot effectively visualizes the classification performance of the model. Greater separation between the two sample groups indicates a more significant classification effect. The first principal component explains 16.60% of the variance, while the second principal component accounts for 13.10%.

#### 3.3.2 Screening of differential plasma metabolites

**Figure 6:**
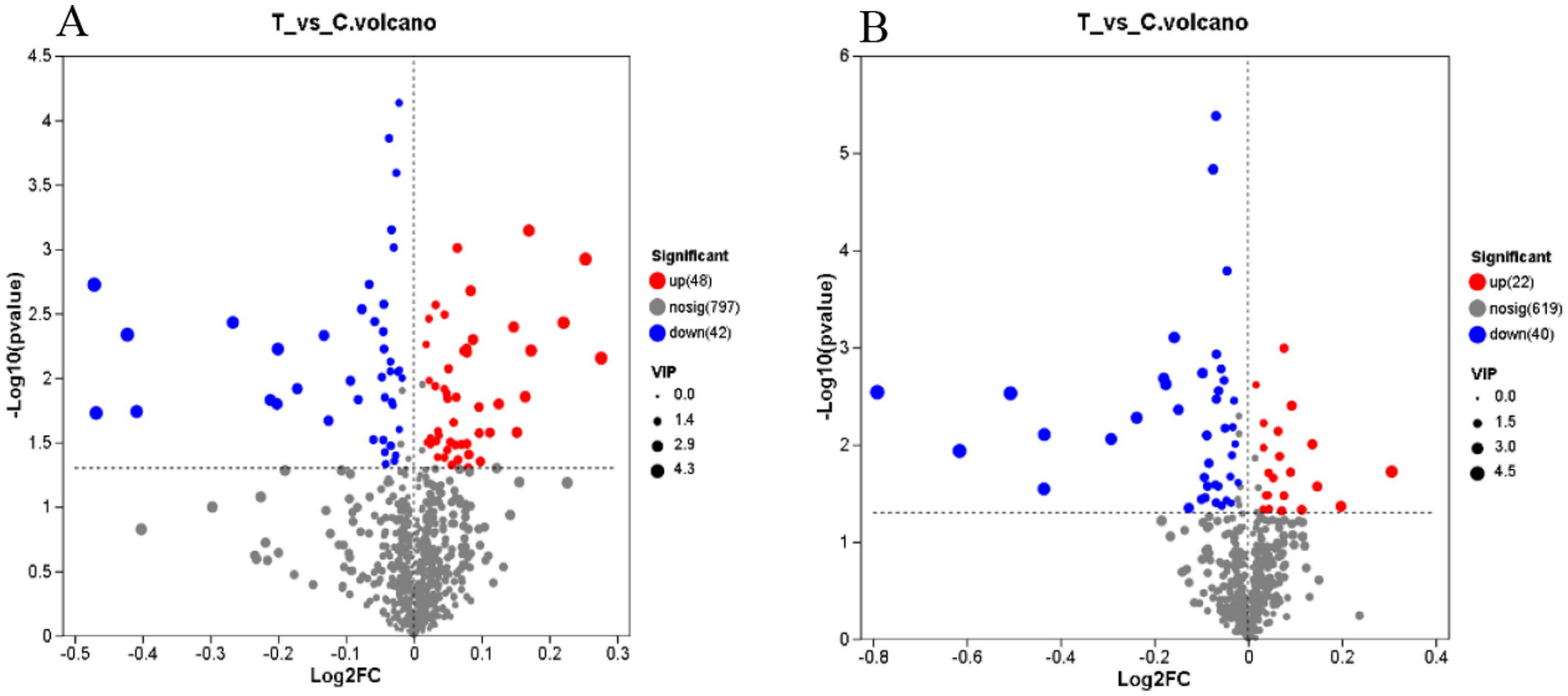
Volcanic diagram of differences in the merging of anions and cations. **(A)** Cation mode **(B)** Anion mode. The horizontal axis represents the fold change in metabolite expression between the two groups, i.e., log2FC, while the vertical axis represents the statistical test value of the difference in metabolite expression levels, i.e., −log10 (*p*_value). Higher values indicate more significant expression differences, and both axes are logarithmically transformed. Each point in the graph represents a specific metabolite, with the size of the point indicating the VIP value. By default, red points denote significantly upregulated metabolites, blue points denote significantly downregulated metabolites, and gray points represent non-significant differences. **(A,B)** C denotes the control group; T represents the experimental group.

Figure 6 highlights a total of 152 differential metabolites identified in both anion and cation modes, with 70 upregulated and 82 downregulated metabolites. The OPLS-DA model, using VIP values > 1 and *p* < 0.05 as the criteria for significant differential metabolites, revealed a total of 50 metabolites in cation mode, 29 of which exhibited an upward trend. In anion mode, 13 metabolites were identified, with 4 showing an upward trend (Supplementary Tables S4, S5).

#### 3.3.3 KEGG pathway enrichment analysis

**Figure 7:**
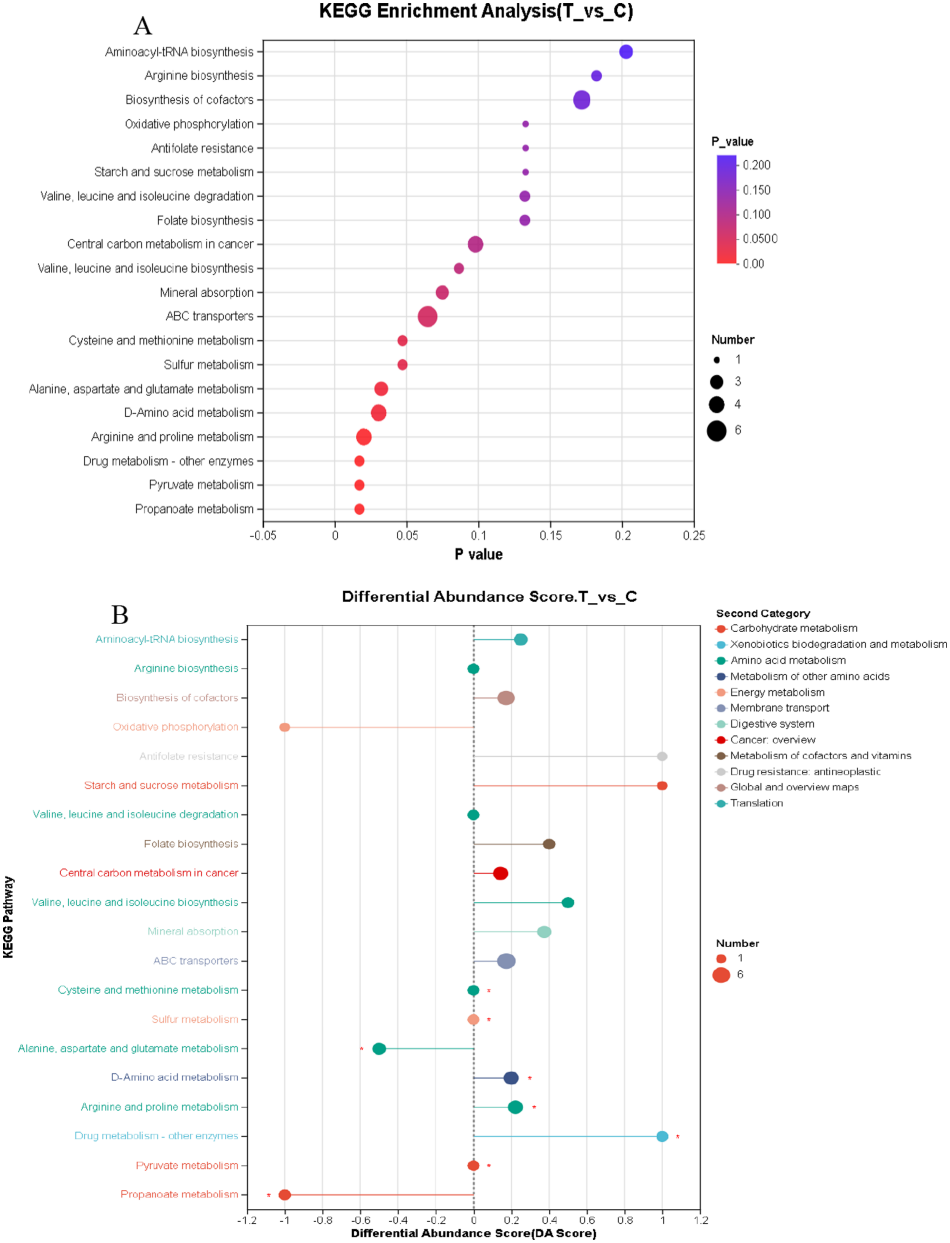
**(A)** KEGG enrichment analysis bubble chart **(B)** KEGG pathway differential abundance score chart. **(A,B)** C denotes the control group; T represents the experimental group. **(A)** The horizontal axis represents the enrichment significance *p*-value; the smaller the p-value, the more statistically significant it is, with a *p*-value less than 0.05 generally considered as a significant enrichment item. The vertical axis represents the KEGG pathways. The size of the bubbles in the graph indicates the number of metabolites enriched in the metabolic set within that pathway. **(B)** In the graph, the horizontal axis represents the Differential Abundance Score (DA Score), while the vertical axis lists the names of the KEGG metabolic pathways. The DA Score reflects the overall changes in all metabolites within a metabolic pathway, with a score of 1 indicating an upward trend in the expression of all annotated differential metabolites in the pathway, and −1 indicating a downward trend. The length of the line segment represents the absolute value of the DA Score. The size of the dots indicates the number of annotated differential metabolites in the pathway, with larger dots representing a greater number of differential metabolites. Dots located to the right of the central axis with longer line segments suggest that the overall expression of the pathway tends to be upregulated; conversely, dots to the left of the central axis with longer line segments indicate a tendency towards downregulation in the pathway’s overall expression.

As presented in Figure 7A, among the top 20 key enriched pathways, 8 metabolic pathways exhibited significant differences (*p* < 0.05), including Propanoate metabolism, Pyruvate metabolism, Drug metabolism – other enzymes, Arginine and proline metabolism, D-Amino acid metabolism, Alanine, aspartate and glutamate metabolism, Sulfur metabolism, and Cysteine and methionine metabolism. These results indicate that a large portion of the identified differential metabolites are closely associated with the biological functions of these 8 metabolic pathways.

#### 3.3.4 Differential abundance in KEGG pathways

Figure 7B identifies 12 metabolic pathways that tend to be upregulated, including Aminoacyl-tRNA biosynthesis, Biosynthesis of cofactors, Antifolate resistance, Starch and sucrose metabolism, Folate biosynthesis, Central carbon metabolism in cancer, Valine, leucine, and isoleucine biosynthesis, Mineral absorption, ABC transporters, D-Amino acid metabolism, Arginine and proline metabolism, and Drug metabolism – other enzymes. The upregulation trends in pathways such as Starch and sucrose metabolism, Antifolate resistance, and Drug metabolism – other enzymes are significantly more pronounced compared to other pathways. Conversely, 3 metabolic pathways exhibited downregulation, namely Oxidative phosphorylation, Alanine, aspartate, and glutamate metabolism, and Propanoate metabolism.

### 3.4 Effects of *L*-Citrulline supplementation on reproductive hormone levels in plasma of Hu sheep ewes

**Figure 8:**
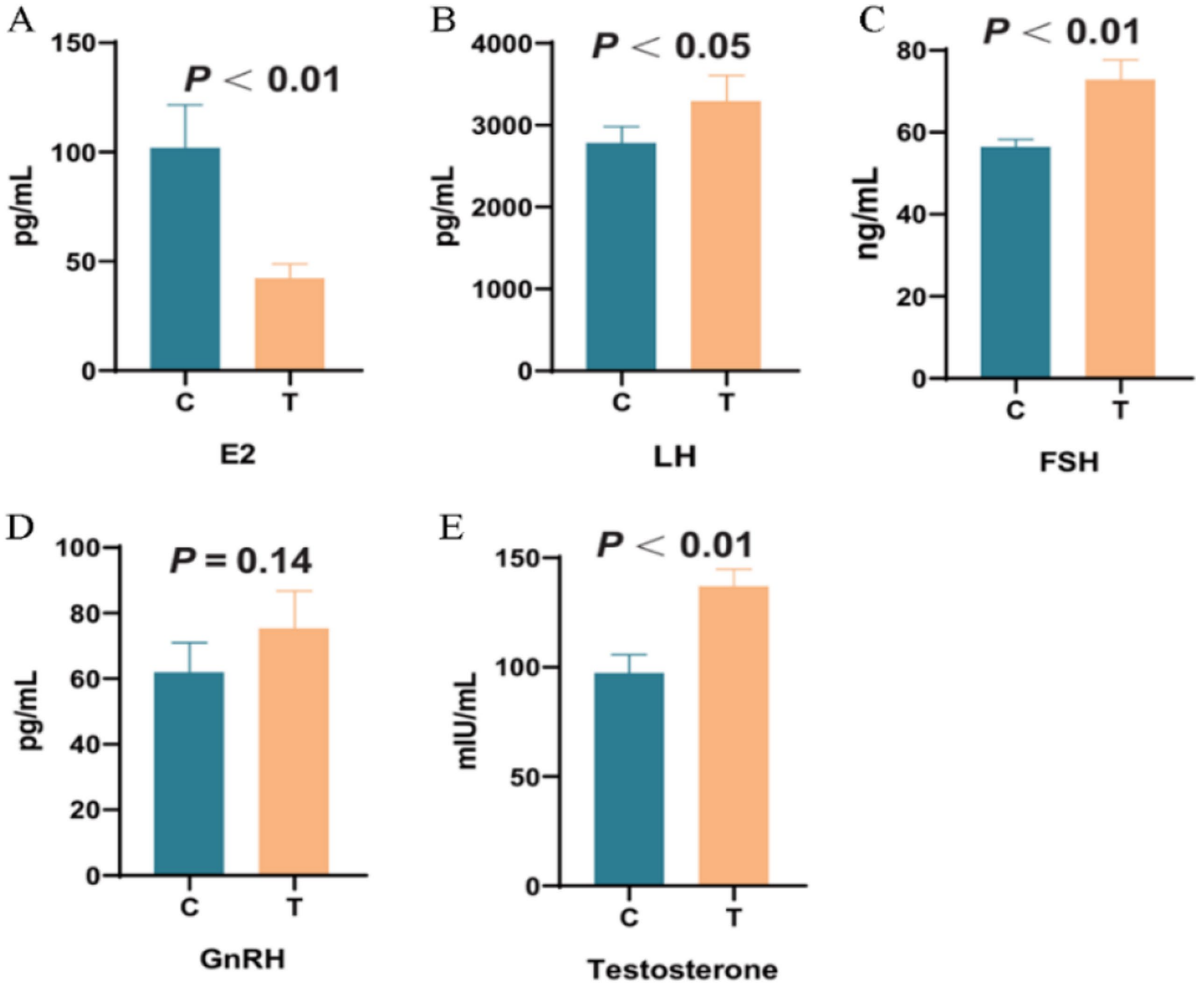
The effect of supplementing *L*-Cit on plasma reproductive hormones. Note: C denotes the control group; T represents the experimental group. **(A)**: Estradiol; **(B)**: Luteinizing hormone; **(C)**: Follicle-stimulating hormone; **(D)**: Gonadotropin-releasing hormone; **(E)**: Testosterone.

As shown in Figure 8, compared with the control group, the plasma E2 level in the experimental group in Figure 8A was significantly reduced by 54.67% (*p* < 0.01). The LH level in Figure 8B was increased by 22.42% (*p* < 0.05), the FSH level in Figure 8C was increased by 16.70% (*p* < 0.05), and the Testosterone level in Figure 8E was increased by 30.02% (*p* < 0.01). No significant change was observed in the GnRH level in Figure 8D.

### 3.5 Effects of *L*-Citrulline supplementation on plasma antioxidant capacity in Hu sheep ewes

**Figure 9:**
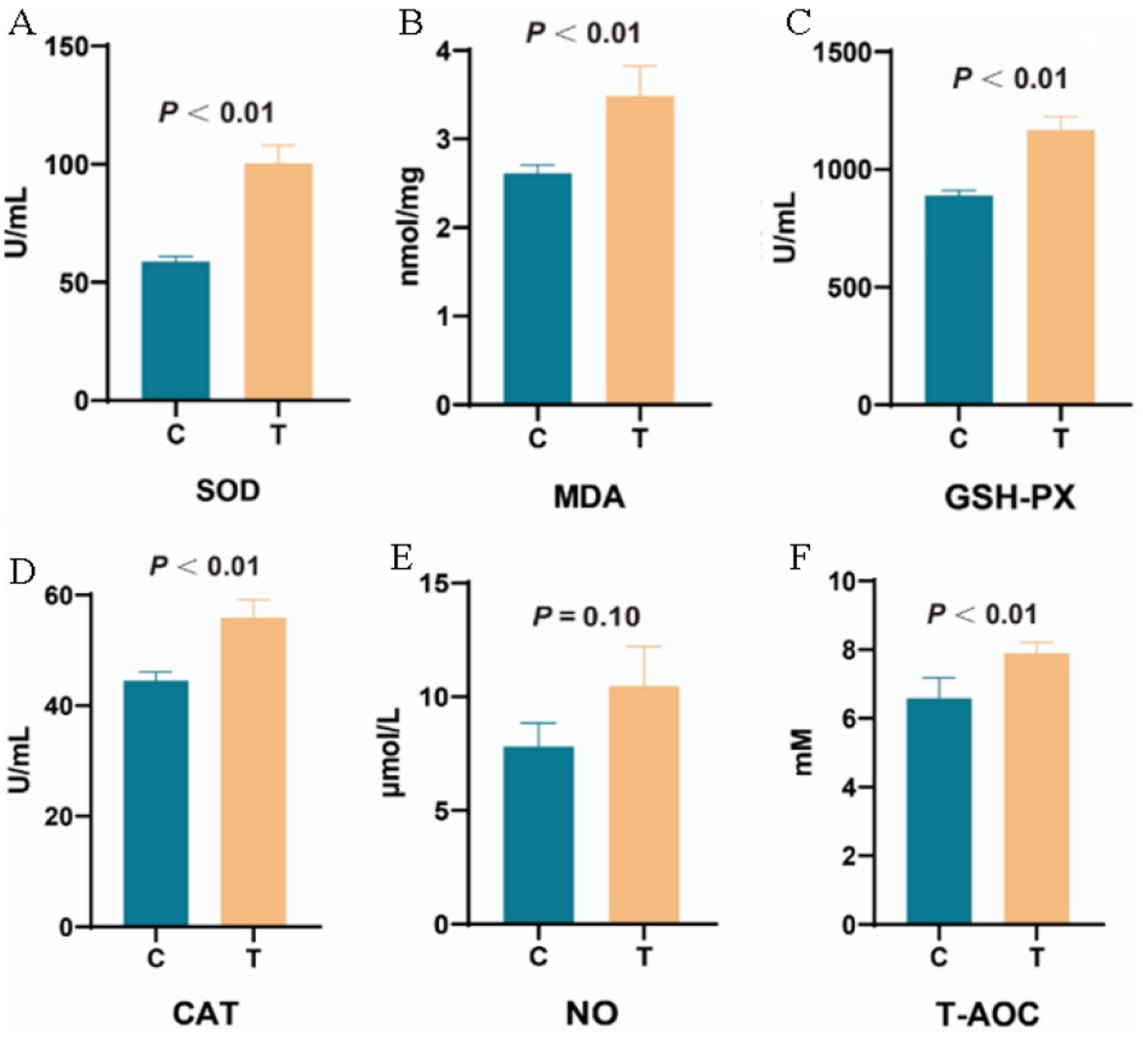
The effect of supplementing *L*-Cit on plasma antioxidant capacity. **(A)** Superoxide Dismutase; **(B)** Malondialdehyde; **(C)** Glutathione Peroxidase; **(D)** Catalase; **(E)** Nitric Oxide; **(F)** Total Antioxidant Capacity. Note: C denotes the control group; T represents the experimental group.

Figure 9 demonstrates that, compared to the Control group, the Experimental group showed significant increases in antioxidant activity: SOD activity increased by 48.30% (*p* < 0.01), MDA levels increased by 23.36% (*p* < 0.01), GSH-PX activity increased by 26.50% (*p* < 0.01), CAT activity increased by 18.49% (*p* < 0.01), and T-AOC increased by 21.43% (*p* < 0.01). However, no significant change in NO levels was observed between the two groups.

### 3.6 Effects of *L*-Citrulline supplementation on estrus rate and conception rate in Hu sheep ewes

On the 30th day after mating, B-ultrasound detection was conducted. After restraining the ewe, the probe was slowly inserted obliquely upwards into the reproductive tract. Upon reaching the uterine position, the probe was aligned vertically and tightly with the coupling agent-coated skin. As the probe moved, the screen image was monitored. Once pregnancy was confirmed, the ewe’s ear tag number was recorded, and the conception rate was calculated. The estrus and conception rates are summarized in Table 2.

**Table 2:** The effect of *L*-Citrulline on estrus rate and conception rate of Hu ewes (%).

Item	Control group (C) group	Experimental group (T) group	*P*-value
Estrus rate	50.00^b^%(15/30)	93.00^a^%(28/30)	<0.01
Conception rate	73.00%(11/15)	82.00%(23/28)	-

## 4 Correlation analysis

### 4.1 Correlation analysis of rumen microbial Flora with plasma reproductive hormones and antioxidant capacity

#### 4.1.1 Correlation analysis between rumen microflora (phylum level) and plasma reproductive hormones and antioxidant capacity

**Figure 10:**
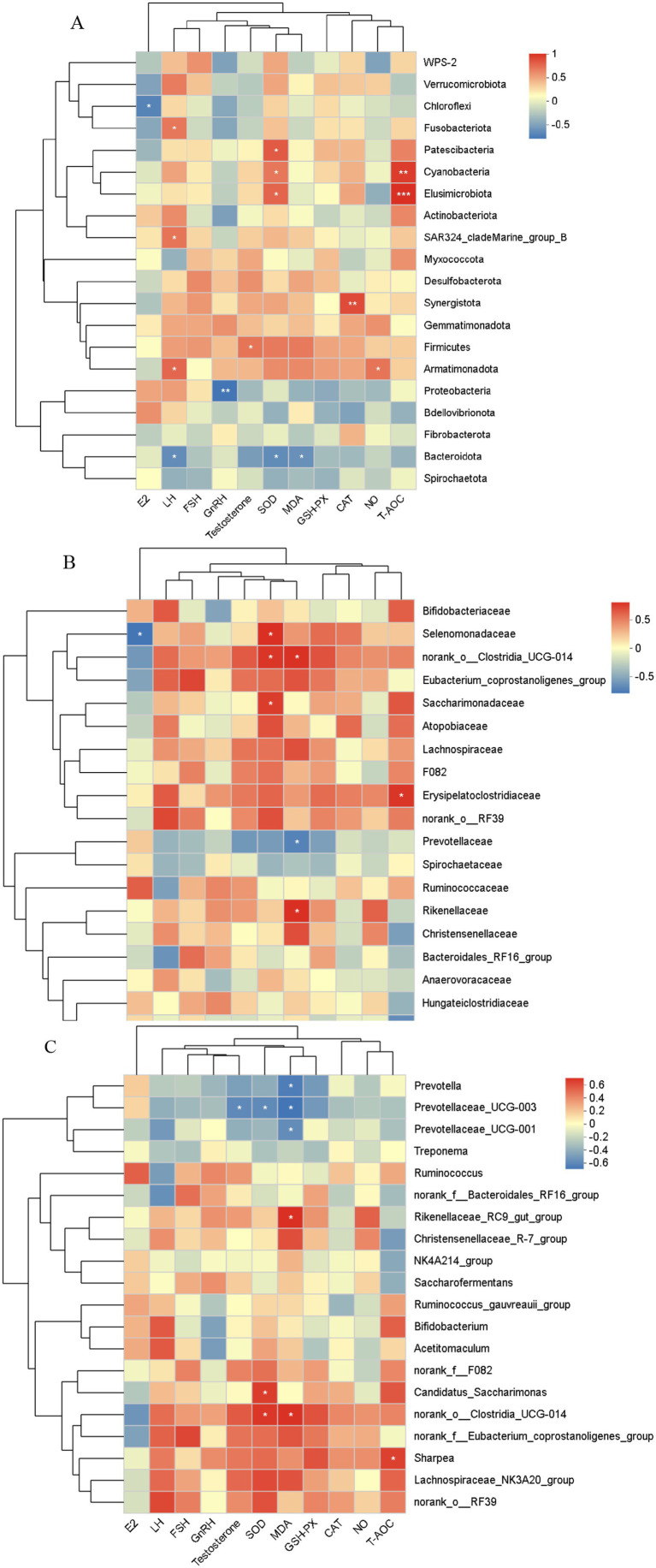
Correlation analysis of rumen microbiota with reproductive hormones and antioxidant markers across different levels. **(A)** Phylum level, **(B)** Family level, **(C)** Genus level. The X-axis and Y-axis represent environmental factors and species, respectively. The *R*-value and *p*-value are calculated. The R-value is displayed in different colors in the graph, and if the p-value is less than 0.05, it is marked with an asterisk (*). The legend on the right shows the color range for different *R*-values. *0.01 < *p* ≤ 0.05, **0.001 < *p* ≤ 0.01, ***p ≤ 0.001.

The correlation heatmap visualizes the relationships between species and environmental factors, offering a clear representation of correlation magnitudes among various environmental factors and species. It also reveals whether these correlations exhibit significant differences. As illustrated in Figure 10A, correlation analysis of the rumen microbiota of Hu sheep ewes at the phylum level and plasma reproductive hormones identified a significant negative correlation between E_2_ levels and Chloroflexi abundance (0.01 < *p* ≤ 0.05). LH levels were significantly positively correlated with the abundances of Fusobacteriota, SAR324_cladeMarine_group_B, and Armatimonadota (0.01 < *p* ≤ 0.05), while negatively correlated with Bacteroidota abundance (0.01 < *p* ≤ 0.05). GnRH content showed a highly significant negative correlation with Proteobacteria (0.001 < *p* ≤ 0.01). T concentration was positively correlated with Firmicutes abundance (0.01 < *p* ≤ 0.05).

As shown in Figure 10A, a correlation analysis of the rumen microbiota at the phylum level and antioxidant capacity revealed that SOD activity was significantly positively correlated with the abundances of Patescibacteria, Cyanobacteria, and Elusimicrobiota (0.01 < *p* ≤ 0.05), while negatively correlated with Bacteroidota (0.01 < *p* ≤ 0.05). MDA levels were negatively correlated with Bacteroidota (0.01 < *p* ≤ 0.05). CAT activity showed a highly significant positive correlation with Synergistota abundance (0.001 < *p* ≤ 0.01). NO concentration was positively correlated with Armatimonadota abundance (0.01 < *p* ≤ 0.05). T-AOC concentration was highly positively correlated with the abundance of Elusimicrobiota and Cyanobacteria (*p* ≤ 0.001; 0.001 < *p* ≤ 0.01).

#### 4.1.2 Correlation analysis between rumen microflora (family level) and plasma reproductive hormones and antioxidant capacity

Figure 10B depicts the correlation analysis of rumen microbiota at the family level and plasma reproductive hormones, revealing a significant negative correlation between E_2_ levels and Selenomonadaceae abundance (0.01 < *p* ≤ 0.05). MDA content was significantly positively correlated with the abundance of norank_o__Clostridia_UCG-014 and Rikenellaceae (0.01 < *p* ≤ 0.05), but negatively correlated with Prevotellaceae abundance (0.01 < *p* ≤ 0.05). T-AOC concentration was significantly positively correlated with Erysipelatoclostridiaceae abundance (0.01 < *p* ≤ 0.05), while negatively correlated with Oscillospiraceae abundance (0.01 < *p* ≤ 0.05).

#### 4.1.3 Correlation analysis between rumen microflora (genus level) and plasma reproductive hormones and antioxidant capacity

As depicted in Figure 10C, correlation analysis of rumen microbiota at the family level and plasma reproductive hormones in Hu sheep ewes revealed a significant negative correlation between T content and Prevotellaceae_UCG-003 abundance (0.01 < *p* ≤ 0.05). Additionally, as shown in Figure 10C, a correlation analysis of rumen microbiota at the phylum level and antioxidant capacity indicated that SOD activity was significantly positively correlated with Candidatus_Saccharimonas and norank_o__Clostridia_UCG-014 abundance (0.01 < *p* ≤ 0.05), but significantly negatively correlated with Prevotellaceae_UCG-003 abundance (0.01 < *p* ≤ 0.05). MDA content was significantly positively correlated with norank_o__Clostridia_UCG-014 abundance (0.01 < *p* ≤ 0.05) and significantly negatively correlated with the abundance of Prevotellaceae_UCG-001, Prevotella, and Prevotellaceae_UCG-003 (0.01 < *p* ≤ 0.05). T-AOC concentration was significantly positively correlated with Sharpea abundance (0.01 < *p* ≤ 0.05).

### 4.2 Correlation analysis between rumen microbiota and volatile fatty acids

#### 4.2.1 Correlation analysis between rumen microbiota (phylum level) and volatile fatty acids

**Figure 11:**
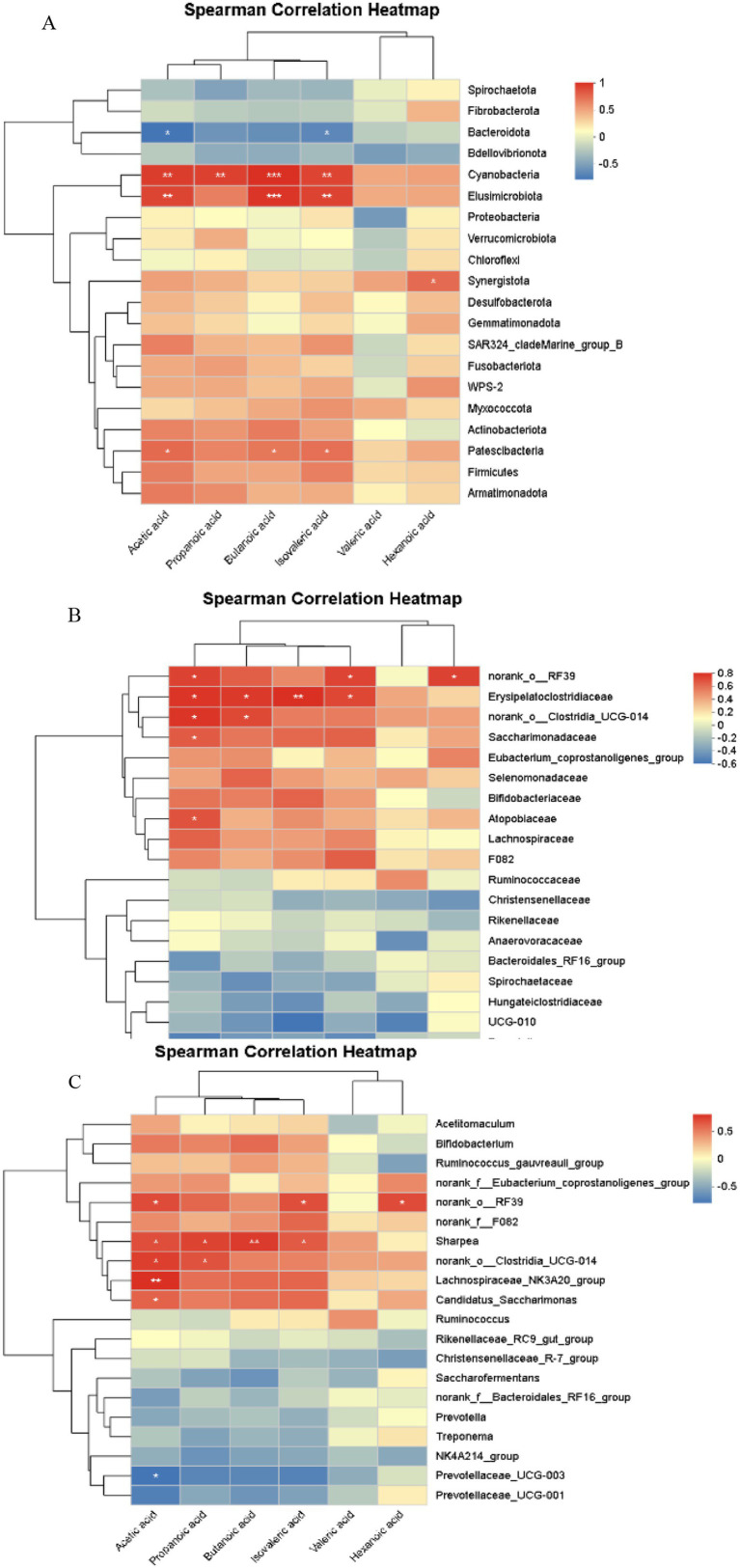
Correlation analysis of rumen microbiota and volatile fatty acids (VFAs) across different levels. **(A)** Phylum level, **(B)** Family level, **(C)** Genus level. The X-axis and Y-axis represent environmental factors and species, respectively. The *R*-value and *p*-value are calculated. The *R*-value is displayed in different colors in the graph, and if the *p*-value is less than 0.05, it is marked with an asterisk (*). The legend on the right shows the color range for different *R*-values. *0.01 < *p* ≤ 0.05, **0.001 < *p* ≤ 0.01, ***p ≤ 0.001.

As shown in Figure 11A, correlation analysis of rumen microbiota at the phylum level and volatile fatty acids (VFAs) in Hu sheep ewes revealed that the relative abundance of acetic acid was significantly positively correlated with Elusimicrobiota and Cyanobacteria (0.001 < *p* ≤ 0.01), as well as with Patescibacteria (0.01 < *p* ≤ 0.05), and significantly negatively correlated with Bacteroidota (0.01 < *p* ≤ 0.05). Propanoic acid abundance showed a significant positive correlation with Cyanobacteria (0.01 < *p* ≤ 0.05). Butanoic acid was significantly positively correlated with Elusimicrobiota and Cyanobacteria (0.001 < *p* ≤ 0.01), and Patescibacteria (0.01 < *p* ≤ 0.05). Isovaleric acid was significantly positively correlated with Elusimicrobiota and Cyanobacteria (0.001 < *p* ≤ 0.01) as well as with Patescibacteria (0.01 < *p* ≤ 0.05), and negatively correlated with Bacteroidota (0.01 < *p* ≤ 0.05). Hexanoic acid abundance showed a significant positive correlation with Synergistota (0.01 < *p* ≤ 0.05).

#### 4.2.2 Correlation analysis between rumen microbiota (family level) and volatile fatty acids

As shown in Figure 11B, correlation analysis of rumen microbiota at the family level and VFAs in Hu sheep ewes demonstrated that acetic acid was significantly positively correlated with norank_o__RF39, Erysipelatoclostridiaceae, norank_o__Clostridia_UCG-014, Saccharimonadaceae, and Atopobiaceae (0.01 < *p* ≤ 0.05). Propanoic acid abundance was significantly positively correlated with Erysipelatoclostridiaceae and norank_o__Clostridia_UCG-014 (0.01 < *p* ≤ 0.05). Butanoic acid was significantly positively correlated with Erysipelatoclostridiaceae (0.001 < *p* ≤ 0.01). Isovaleric acid was significantly positively correlated with norank_o__RF39 and Erysipelatoclostridiaceae (0.01 < *p* ≤ 0.05). Hexanoic acid abundance showed a significant positive correlation with norank_o__RF39 (0.01 < *p* ≤ 0.05).

#### 4.2.3 Correlation analysis between rumen microbiota (genus level) and volatile fatty acids

As shown in Figure 11C, correlation analysis of rumen microbiota at the genus level and VFAs in Hu sheep ewes revealed that the relative abundance of acetic acid was significantly positively correlated with Lachnospiraceae_NK3A20_group (0.001 < *p* ≤ 0.01), and with norank_o_RF39, Sharpea, norank_o_Clostridia_UCG-014, and Candidatus_Saccharimonas (0.01 < *p* ≤ 0.05). It was significantly negatively correlated with the abundance of Prevotellaceae_UCG-003 (0.01 < *p* ≤ 0.05). Propanoic acid abundance was significantly positively correlated with Sharpea and norank_o_Clostridia_UCG-014 (0.01 < *p* ≤ 0.05). Butanoic acid was significantly positively correlated with Sharpea (0.001 < *p* ≤ 0.01). Isovaleric acid abundance was significantly positively correlated with norank_o_RF39 and Sharpea (0.01 < *p* ≤ 0.05). Hexanoic acid abundance was significantly positively correlated with norank_o_RF39 (0.01 < *p* ≤ 0.05).

### 4.3 Correlation analysis of plasma metabolites with reproductive hormones and antioxidant capacity

**Figure 12:**
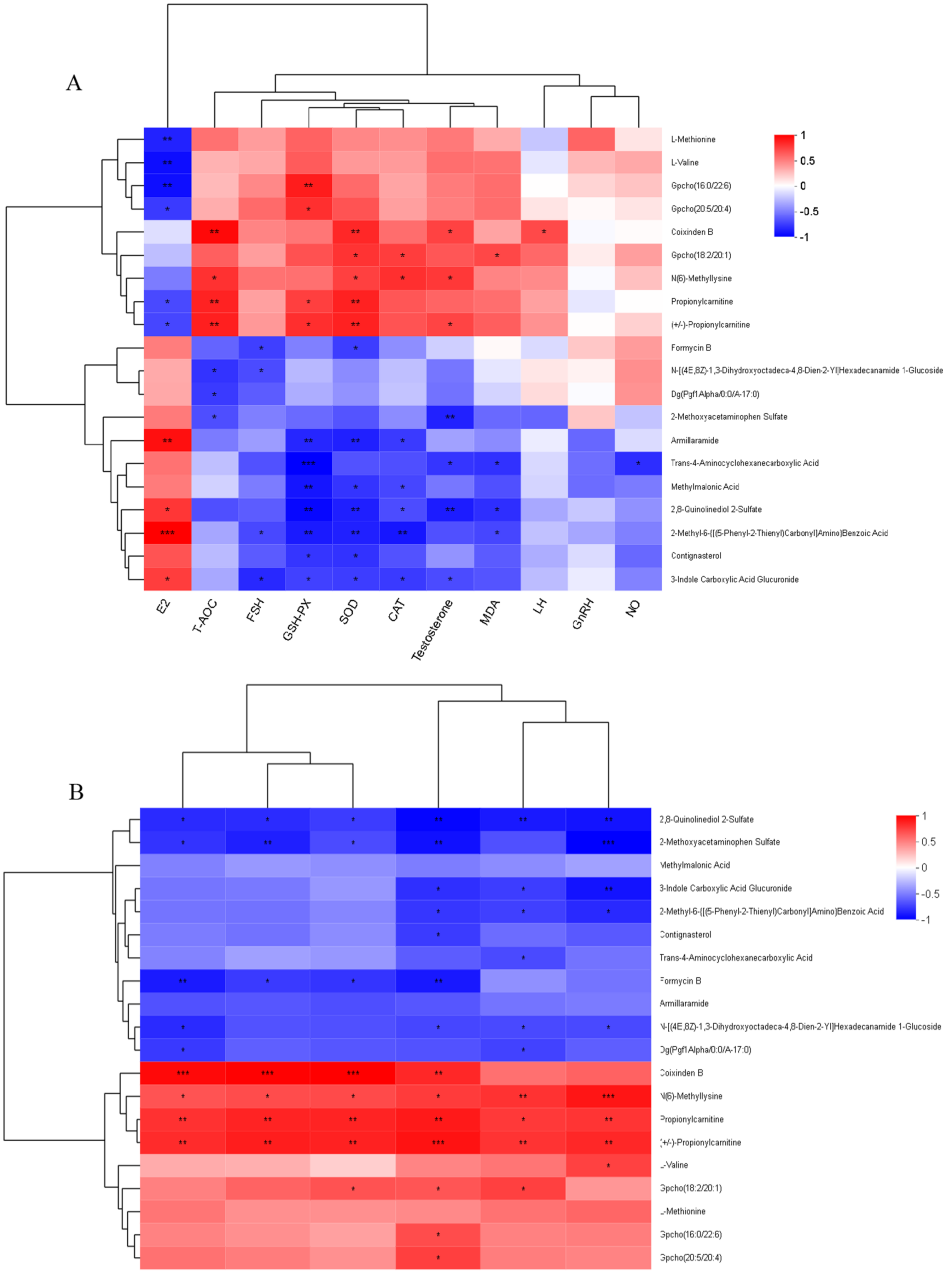
**(A)** Correlation network: plasma metabolites vs. reproductive hormones and antioxidant markers. **(B)** Correlation analysis plot of plasma differential metabolites and volatile fatty acids. The right side of the graph displays the names of metabolites, while the bottom lists the names of associated data. Each cell in the graph represents the correlation between two attributes (metabolites and associated features), with different colors indicating the magnitude of the correlation coefficient between the attributes. Asterisks denote the significance level of the p-value: *0.01 < *p* ≤ 0.05, **0.001 < *p* ≤ 0.01, ***p ≤ 0.001.

As shown in Figure 12A, correlation analysis of plasma differential metabolites (top 20) and plasma reproductive hormones in Hu sheep ewes revealed that E_2_ levels were positively correlated with 2-Methyl-6-{[(5-Phenyl-2-Thienyl)Carbonyl]Amino} Benzoic Acid, Armillaramide, 2,8-Quinolinediol 2-Sulfate, and 3-Indole Carboxylic Acid Glucuronide (*p* ≤ 0.001, 0.001 < *p* ≤ 0.01, 0.01 < *p* ≤ 0.05), and negatively correlated with *L*-Methionine, *L*-Valine, Gpcho (16:0/22:6), Gpcho (20:5/20:4), Propionylcarnitine, and (+/−)-Propionylcarnitine (0.001 < *p* ≤ 0.01, 0.01 < *p* ≤ 0.05). LH levels were positively correlated with Coixinden B (0.01 < *p* ≤ 0.05). FSH levels were negatively correlated with Formycin B, N-[(4E,8Z)-1,3-Dihydroxyoctadeca-4,8-Dien-2-Yl] Hexadecanamide 1-Glucoside, 2-Methyl-6-{[(5-Phenyl-2-Thienyl)Carbonyl]Amino} Benzoic Acid, and 3-Indole Carboxylic Acid Glucuronide (0.01 < *p* ≤ 0.05). T levels were positively correlated with Coixinden B, N (6)-Methyllysine, and (+/−)-Propionylcarnitine (0.01 < *p* ≤ 0.05), and negatively correlated with 2-Methoxyacetaminophen Sulfate, 2,8-Quinolinediol 2-Sulfate, Trans-4-Aminocyclohexanecarboxylic Acid, and 3-Indole Carboxylic Acid Glucuronide (0.001 < *p* ≤ 0.01, 0.01 < *p* ≤ 0.05).

As shown in Figure 12A, correlation analysis of plasma differential metabolites (top 20) and antioxidant indicators in Hu sheep ewes revealed that SOD activity was significantly positively correlated with Coixinden B, Propionylcarnitine, and (+/−)-Propionylcarnitine (0.001 < *p* ≤ 0.01), and with Gpcho (18:2/20:1) and N (6)-Methyllysine (0.01 < *p* ≤ 0.05). It was significantly negatively correlated with Armillaramide, 2,8-Quinolinediol 2-Sulfate, and 2-Methyl-6-{[(5-Phenyl-2-Thienyl)Carbonyl]Amino} Benzoic Acid (0.001 < *p* ≤ 0.01), as well as with Formycin B, Methylmalonic Acid, Contignasterol, and 3-Indole Carboxylic Acid Glucuronide (0.01 < *p* ≤ 0.05).

MDA content exhibited a significant positive correlation with Gpcho (18:2/20:1) (0.01 < *p* ≤ 0.05) and a significant negative correlation with Trans-4-Aminocyclohexanecarboxylic Acid, 2,8-Quinolinediol 2-Sulfate, and 2-Methyl-6-{[(5-Phenyl-2-Thienyl)Carbonyl]Amino} Benzoic Acid (0.01 < *p* ≤ 0.05).

GSH-PX was significantly positively correlated with Gpcho (16:0/22:6) (0.001 < *p* ≤ 0.01); Gpcho (20:5/20:4), Propionylcarnitine, and (+/−)-Propionylcarnitine (0.01 < *p* ≤ 0.05); and significantly negatively correlated with Trans-4-Aminocyclohexanecarboxylic Acid (*p* ≤ 0.001), Armillaramide, Methylmalonic Acid, 2,8-Quinolinediol 2-Sulfate, and 2-Methyl-6-{[(5-Phenyl-2-Thienyl)Carbonyl]Amino} Benzoic Acid (0.001 < *p* ≤ 0.01). Additionally, it exhibited significant negative correlations with Contignasterol and 3-Indole Carboxylic Acid Glucuronide (0.01 < *p* ≤ 0.05).

CAT showed a significant positive correlation with Gpcho (18:2/20:1) and N (6)-Methyllysine (0.01 < *p* ≤ 0.05), and a significant negative correlation with 2-Methyl-6-{[(5-Phenyl-2-Thienyl)Carbonyl]Amino} Benzoic Acid (0.001 < *p* ≤ 0.01), Armillaramide, Methylmalonic Acid, 2,8-Quinolinediol 2-Sulfate, and 3-Indole Carboxylic Acid Glucuronide (0.01 < *p* ≤ 0.05). NO displayed a significant negative correlation with Trans-4-Aminocyclohexanecarboxylic Acid (0.01 < *p* ≤ 0.05).

T-AOC was extremely positively correlated with Coixinden B, Propionylcarnitine, and (+/−)-Propionylcarnitine (0.001 < *p* ≤ 0.01), positively correlated with N (6)-Methyllysine (0.01 < *p* ≤ 0.05), and negatively correlated with N-[(4E,8Z)-1,3-Dihydroxyoctadeca-4,8-Dien-2-Yl] Hexadecanamide 1-Glucoside, Dg (Pgf1Alpha/0:0/A-17:0), and 2-Methoxyacetaminophen Sulfate (0.01 < *p* ≤ 0.05).

### 4.4 Correlation analysis between plasma metabolites and volatile fatty acids

Figure 12B illustrates the correlation analysis of plasma differential metabolites (top 20) and VFAs in Hu sheep ewes, revealing that the relative abundance of acetic acid was significantly positively correlated with Coixinden B (*p* ≤ 0.001), Propionylcarnitine, and (+/−)-Propionylcarnitine (0.001 < *p* ≤ 0.01), positively correlated with N(6)-Methyllysine (0.01 < *p* ≤ 0.05), and negatively correlated with 2-Methoxyacetaminophen Sulfate (0.001 < *p* ≤ 0.01). Additionally, it showed a significant negative correlation with 2,8-Quinolinediol 2-Sulfate and Formycin B (0.01 < *p* ≤ 0.05).

Propanoic acid demonstrated a significant positive correlation with (+/−)-Propionylcarnitine (*p* ≤ 0.001), Coixinden B, and Propionylcarnitine (0.001 < *p* ≤ 0.01), positively correlated with Gpcho(18:2/20:1), Gpcho(16:0/22:6), and Gpcho(20:5/20:4) (0.01 < *p* ≤ 0.05), and negatively correlated with 2,8-Quinolinediol 2-Sulfate, 2-Methoxyacetaminophen Sulfate, and Formycin B (0.001 < *p* ≤ 0.01). It also exhibited significant negative correlations with 3-Indole Carboxylic Acid Glucuronide, 2-Methyl-6-{[(5-Phenyl-2-Thienyl)Carbonyl]Amino}Benzoic Acid, Contignasterol, and N-[(4E,8Z)-1,3-Dihydroxyoctadeca-4,8-Dien-2-Yl]Hexadecanamide 1-Glucoside (0.01 < *p* ≤ 0.05).

The relative abundance of butanoic acid was significantly positively correlated with Coixinden B (*p* ≤ 0.001), Propionylcarnitine, and (+/−)-Propionylcarnitine (0.001 < *p* ≤ 0.01), as well as with N(6)-Methyllysine and Gpcho(18:2/20:1) (0.01 < *p* ≤ 0.05). It was significantly negatively correlated with 2,8-Quinolinediol 2-Sulfate, 2-Methoxyacetaminophen Sulfate, and Formycin B (0.01 < *p* ≤ 0.05).

Isovaleric acid showed a significant positive correlation with Coixinden B (*p* ≤ 0.001), Propionylcarnitine, and (+/−)-Propionylcarnitine (0.001 < *p* ≤ 0.01), as well as with N(6)-Methyllysine (0.01 < *p* ≤ 0.05). It exhibited significant negative correlations with Formycin B (0.001 < *p* ≤ 0.01), 2,8-Quinolinediol 2-Sulfate, 2-Methoxyacetaminophen Sulfate, N-[(4E,8Z)-1,3-Dihydroxyoctadeca-4,8-Dien-2-Yl]Hexadecanamide 1-Glucoside, and Dg(Pgf1Alpha/0:0/A-17:0) (0.01 < *p* ≤ 0.05).

Valeric acid was significantly positively correlated with N(6)-Methyllysine and (+/−)-Propionylcarnitine (0.001 < *p* ≤ 0.01), Propionylcarnitine, and Gpcho(18:2/20:1) (0.01 < *p* ≤ 0.05). It showed a significant negative correlation with 2,8-Quinolinediol 2-Sulfate (0.001 < *p* ≤ 0.01) and with 3-Indole Carboxylic Acid Glucuronide, 2-Methyl-6-{[(5-Phenyl-2-Thienyl)Carbonyl]Amino}Benzoic Acid, Trans-4-Aminocyclohexanecarboxylic Acid, N-[(4E,8Z)-1,3-Dihydroxyoctadeca-4,8-Dien-2-Yl]Hexadecanamide 1-Glucoside, and Dg(Pgf1Alpha/0:0/A-17:0) (0.01 < *p* ≤ 0.05).

Hexanoic acid displayed a significant positive correlation with N(6)-Methyllysine (*p* ≤ 0.001), Propionylcarnitine, and (+/−)-Propionylcarnitine (0.001 < *p* ≤ 0.01), as well as with Gpcho(18:2/20:1) (0.01 < *p* ≤ 0.05). It was significantly negatively correlated with 2-Methoxyacetaminophen Sulfate (*p* ≤ 0.001), 2,8-Quinolinediol 2-Sulfate, and 3-Indole Carboxylic Acid Glucuronide (0.001 < *p* ≤ 0.01), and with 2-Methyl-6-{[(5-Phenyl-2-Thienyl)Carbonyl]Amino}Benzoic Acid and N-[(4E,8Z)-1,3-Dihydroxyoctadeca-4,8-Dien-2-Yl]Hexadecanamide 1-Glucoside (0.01 < *p* ≤ 0.05).

## 5 Discussion

The rumen of ruminants, a highly intricate micro-ecosystem, harbors a diverse array of microorganisms, including bacteria, archaea, fungi, and protozoa (Indugu et al., 2017). These microorganisms are essential for maintaining rumen homeostasis, promoting fiber degradation, and regulating nutrient metabolism through a dynamic balance of community structure and relative abundance (Stewart et al., 2018). Specifically, plant cell wall polysaccharides are broken down by rumen microbes, releasing VFA, microbial proteins, vitamins, and other nutrients via the secretion of hydrolytic enzymes such as cellulases and hemicellulases. This process provides approximately 70% of the host’s energy requirements (Wirth et al., 2018). Microbial composition analysis reveals that the phyla Firmicutes and Bacteroidetes dominate the rumen microbiome, accounting for over 80% of the microbial community. Core functional genera, including Prevotella, Ruminococcus, and Succinivibrio, collaborate in the cascade of fiber degradation through synergistic interactions (Calabrò, 2015). The metabolic pathways involved include the depolymerization of cellulose into oligosaccharides by primary degraders, followed by conversion into intermediates like succinate and lactate by secondary degraders. These intermediates are subsequently converted into VFAs (acetate:propionate:butyrate ≈ 70:20:10) by acetate-producing and methane-producing bacteria (Stevens and Hume, 1998). In this study, *L*-Cit supplementation significantly altered the rumen microbial community structure. At the phylum level, the relative abundance of Firmicutes increased significantly (*p* < 0.05), while Bacteroidetes and Proteobacteria abundances significantly decreased (*p* < 0.05). Notably, the reduction in Bacteroidetes, which play a pivotal role in soluble polysaccharide metabolism, may be linked to *L*-Cit’s regulation of carbohydrate-active enzyme expression (Thomas et al., 2011). Concurrently, the upregulation of genes involved in butyrate synthesis within the Firmicutes phylum may explain the observed increase in butyrate proportion among the VFAs. At the genus level, an increase in Ruminococcus and a decrease in Prevotella abundance were detected in the Experimental group. Additionally, the reduced abundance of Proteobacteria may reflect the inhibition of opportunistic pathogens by *L*-Cit through modulation of redox status (Moon et al., 2018). Alpha diversity analysis revealed higher Sobs, Chao1, and Ace indices in the Experimental group compared to the control group, suggesting that *L*-Cit enhances microbial community diversity. This increase in diversity could be attributed to *L*-Cit’s role as an arginine precursor, facilitating nitrogen metabolism within the microbial community (Gilbreath et al., 2020).

This study conducted a systematic analysis of the correlations between the rumen microbiota of Hu sheep ewes and plasma reproductive hormones as well as antioxidant capacity indicators at the phylum, family, and genus levels, uncovering potential links between microbial composition and host physiological functions. The key findings at each taxonomic level are as follows: at the phylum level, the significant negative correlation between Chloroflexi and E_2_ suggests that this phylum may inhibit estrogen activity through metabolic or signaling pathways, although the exact mechanisms warrant further investigation. The positive correlations between Fusobacteriota and Armatimonadota with LH may be linked to their role in VFA metabolism. Additionally, the positive correlation between Firmicutes and T could be attributed to the influence of this phylum on steroid hormone synthesis via bile acid metabolism (Lee et al., 2022). In terms of antioxidant capacity, the positive correlations of Cyanobacteria and Elusimicrobiota with SOD activity and T-AOC may reflect their secretion of antioxidant enzymes or the antioxidant properties of their metabolites. The negative correlations between Bacteroidota and both SOD activity and MDA suggest that this phylum might affect oxidative balance by promoting oxidative stress or inhibiting host antioxidant enzyme activity. At the family level, Selenomonadaceae was negatively correlated with E_2_ but positively correlated with SOD activity, indicating that this family of microorganisms might influence hormone metabolism and redox balance through distinct mechanisms. At the genus level, Prevotellaceae_UCG-003 showed negative correlations with T and SOD activity, possibly reflecting the inhibitory effects of its fermentation metabolites on hormone synthesis or oxidative stress. Additionally, the positive correlation between norank_o__Clostridia_UCG-014 and MDA suggests that it may exacerbate oxidative damage via lipid peroxidation pathways. This study identified highly significant correlations, such as the association between CAT activity and Synergistota (*p* ≤ 0.001), highlighting the reliability of these results and suggesting potent interactions between the microbiota and host. The functional predictions presented in the manuscript are inherently constrained by the absence of experimental validation, resulting in defined limitations within this section. In conclusion, significant associations were found between the rumen microbiota of Hu sheep and reproductive hormones as well as antioxidant capacity across multiple taxonomic levels, suggesting that the microbiota may regulate reproductive endocrinology and oxidative homeostasis through metabolites or host–microbe interactions.

Rumen VFAs, as essential signaling molecules in rumen microbial metabolism, are intimately linked to the physiological functions of the rumen in ruminants, with changes in their composition being of significant importance (Song et al., 2025). As the end products of carbohydrate and amino acid fermentation, VFAs provide 70–80% of the energy requirements for ruminants, contributing to approximately two-thirds of the body’s metabolic carbon flux (Zhao et al., 2022a), while also regulating rumen environmental homeostasis through concentration gradients. In this study, rumen pH in both the *L*-Cit supplemented Experimental group and the Control group remained within the physiological range (5.5–7.5), with no significant differences observed between the groups (*p* > 0.05), indicating that *L*-Cit supplementation did not negatively impact rumen fermentation in Hu sheep ewes. Analysis of VFA composition revealed specific metabolic changes in the Experimental group: the relative abundances of butyrate (*p* < 0.05) and valerate (*p* < 0.05) were significantly increased, while acetate, propionate, isovalerate, and caproate showed highly significant increases (*p* < 0.01). These alterations were closely associated with changes in the rumen microbial community structure: the relative abundance of Firmicutes (the primary group of fiber-degrading bacteria) increased, whereas the abundance of Bacteroidetes (dominant polysaccharide-degrading bacteria) decreased. Notably, the non-dominant phyla Cyanobacteria and Elusimicrobiota were significantly enriched in the Experimental group (*p* < 0.05) and were extremely significantly positively correlated with acetic acid, butyric acid, and isovaleric acid (*p* < 0.01), suggesting their potential synergistic role in VFA synthesis. Functional analysis indicated that changes in VFA concentrations induced by *L*-Cit have several physiological implications: the increase in butyric acid enhances the energy supply to ruminal epithelial cells (Fu et al., 2022), while the accumulation of isovaleric acid improves microbial protein efficiency by promoting the synthesis of branched-chain amino acids (Zhang et al., 2022). More importantly, the elevated VFA concentrations activated the host’s immune regulatory network: intestinal mucosal dendritic cells and macrophages were stimulated via the GPCR pathway (Shi et al., 2017), leading to the promotion of anti-inflammatory cytokine secretion. Simultaneously, the chemotactic activity of neutrophils was enhanced (Dahlstrand Rudin et al., 2021; Miller et al., 2023), thereby establishing a positive regulation of the “microbiota-metabolite-immunity” axis.

This study analyzed the associations between ruminal microbiota and VFAs in Hu sheep ewes supplemented with *L*-Cit, uncovering the complex relationships between microbial community structure at different taxonomic levels (phylum, family, genus) and VFA production. At the phylum level, Elusimicrobiota and Cyanobacteria were significantly positively correlated with the abundances of VFAs such as acetic acid, butyric acid, and isovaleric acid (*p* ≤ 0.01), suggesting that these bacterial groups play a pivotal role in VFA synthesis. Additionally, the negative correlation between Bacteroidota and acetic acid as well as isovaleric acid (*p* ≤ 0.05) indicates that this phylum may inhibit VFA metabolic pathways. At the family and genus levels, microbial groups such as Erysipelatoclostridiaceae, Sharpea, and norank_o_RF39 were significantly positively correlated with multiple VFAs, suggesting that these families and genera directly contribute to VFA synthesis. The significant positive correlation between Lachnospiraceae_NK3A20_group and acetic acid (*p* ≤ 0.01) further highlights the important role of Firmicutes members in acetic acid production. In conclusion, the associations between ruminal microbiota and VFAs in Hu sheep ewes were taxonomically specific and potentially regulated by *L*-Cit intervention.

Using non-targeted metabolomics technology, the effects of *L*-Cit supplementation on plasma metabolites in Hu sheep ewes were systematically analyzed, revealing potential metabolic regulatory mechanisms. PLS-DA modeling showed a clear separation between the Control group and the Experimental group, indicating that *L*-Cit intervention significantly influenced plasma metabolite profiles. This finding aligns with the strengths of non-targeted metabolomics in detecting dynamic metabolic changes (Ribbenstedt et al., 2018) and provides reliable evidence for the identification of functional metabolites. A total of 152 significantly differential metabolites were identified (50 in the positive ion mode and 13 in the negative ion mode), with 70 metabolites up-regulated and 82 down-regulated. These results suggest that the biological effects of *L*-Cit are mediated through bidirectional regulation of the metabolic network. Notably, the significant enrichment of arginine and proline metabolic pathways (VIP > 1, *p* < 0.05) is likely linked to their roles in nitrogen metabolism, immune regulation, and muscle synthesis (Majumdar et al., 2016). The significant upregulation of drug metabolic pathways, including the “drug metabolism - other enzymes” pathway, suggests that *L*-Cit may indirectly regulate the metabolic efficiency of exogenous substances by influencing the activity of drug-metabolizing enzymes. This finding could provide a valuable reference for future nutritional interventions combined with drug therapies (Fukami et al., 2022). Differential abundance analysis of KEGG pathways revealed that 12 pathways, such as aminoacyl-tRNA biosynthesis and starch and sucrose metabolism, were significantly upregulated, while 3 pathways, including oxidative phosphorylation, were significantly downregulated. The upregulation of aminoacyl-tRNA biosynthesis suggests enhanced protein translation efficiency, thereby supporting anabolic metabolism in the body (Yoon et al., 2024). Conversely, the downregulation of oxidative phosphorylation may indicate a shift in energy metabolism towards glycolysis (Wilson, 2017).

These findings align with the hypothesis that *L*-Cit, as an NO precursor, may regulate mitochondrial function and redox balance (Dawoud and Malinski, 2020). Notably, the bidirectional changes in D-amino acid metabolism pathways, observed in both enrichment and differential abundance analyses, suggest a complex role in host–microbe interactions, warranting further investigation.

The study also explored the regulatory mechanism of *L*-Cit on reproductive hormones in Hu sheep ewes through the NO metabolic pathway. As an arginine precursor for NO synthesis, *L*-Cit influences NO metabolism in macrophages by regulating the activities of arginase and inducible nitric oxide synthase (iNOS)(Romero et al., 2006). Notably, the NO signaling pathway can act on the hypothalamic–pituitary-gonadal (HPG) axis, regulating the pulsatile secretion of GnRH and the release of FSH and LH from the pituitary, ultimately modulating ovarian steroid hormone synthesis (Wu et al., 2005; Zhang, 2011). The experimental results indicated that *L*-Cit supplementation significantly increased the plasma levels of LH, FSH, and T in Hu sheep ewes (*p* < 0.05), while E_2_ concentration was extremely significantly decreased (*p* < 0.01). These findings align with those reported by Zhao et al. (2022b) in rams but contrast with the results of Ma et al. (2023). The discrepancy may be attributed to factors such as the method of *L*-Cit administration, the timing of intervention, seasonal influences on the endocrine system, and differences in the pretreatment physiological state of the experimental animals. From a mechanistic perspective, the decrease in E_2_ levels may be due to the dual regulatory role of NO: on one hand, NO could reduce gonadotropin release by inhibiting GnRH secretion (Wu et al., 2005), and on the other, it may directly inhibit the activity of aromatase in ovarian granulosa cells (Zhang, 2011). The study also found a significant increase in the estrus rate in the Experimental group, suggesting that *L*-Cit may enhance follicle development and ovulation by optimizing the signaling efficiency of the hypothalamic–pituitary-ovarian axis.

Reactive oxygen species (ROS) are highly reactive chemical species derived from oxygen-containing molecules. They are characterized by the presence of unpaired electrons or unstable chemical bonds and primarily include hydrogen peroxide (H_2_O_2_), superoxide anion (O_2_^−^), hydroxyl radical (-OH), and peroxyl radicals (Shields et al., 2021). These molecules can damage biological macromolecules such as lipids, proteins, carbohydrates, and nucleic acids through oxidative stress. To maintain redox homeostasis, animal organisms rely on an antioxidant defense system, which includes enzymes like SOD, CAT, and GSH-Px. In this study, plasma antioxidant indices in Hu sheep ewes revealed that *L*-Cit supplementation significantly increased the activities of SOD, GSH-Px, and CAT (*p* < 0.01), while the levels of MDA and T-AOC were also significantly higher (*p* < 0.01). Notably, while NO levels showed an increasing trend, the differences in NO levels between groups were not statistically significant. These findings contradict the reported decreases in MDA levels observed in studies by Liu (2021) and Li et al. (2024), suggesting that oxidative stress processes may be regulated by *L*-Cit through specific mechanisms. Nitric oxide (NO) exhibits a marked concentration-dependent biphasic effect in biological systems: antioxidative protection is mediated through free radical scavenging at low concentrations, while peroxynitrite (ONOO^−^), a potent oxidant, is generated via reaction with superoxide anion (O₂^−^) at elevated concentrations, leading to oxidative damage such as lipid peroxidation (He et al., 2010). This dual mechanism is manifested through two distinct pathways by which cellular functions are modulated: biological macromolecules are modified either through direct oxidative reactions mediated by ONOO^−^, or cellular structures are disrupted via free radical-mediated indirect mechanisms. Consequently, signal fine-tuning is achieved under physiological conditions, while severe oxidative stress is induced during pathological states (Pacher et al., 2007). As central mediators in inflammatory regulation, immune responses are coordinated through a cascade signaling network by inflammatory cytokines. Attenuated inflammatory reactions are directly associated with reduced expression levels of these cytokines (Hunter and Jones, 2015). Specifically, bovine mammary tissue damage is demonstrated to be alleviated and milk production performance is significantly enhanced through inhibition of key factors such as IL-1β (Alnakip et al., 2014). Under heat stress conditions, the upregulation of IL-1β and IL-6 expression is effectively suppressed through supplementation with antioxidants such as vitamin E, resulting in dairy cow production performance being consequently improved (Molinari et al., 2023). Notably, in the weaned calf model, a reduction in diarrhea incidence and a 23% enhancement in daily weight gain are achieved through downregulation of inflammatory cytokines (Hulbert and Moisá, 2016). Such precise regulation is mediated by a dynamic equilibrium network composed of cytokines, their inhibitory factors, and soluble receptors, where spatiotemporal modulation of inflammatory progression is achieved through multi-target synergy (Vebr et al., 2023).

**Figure 13:**
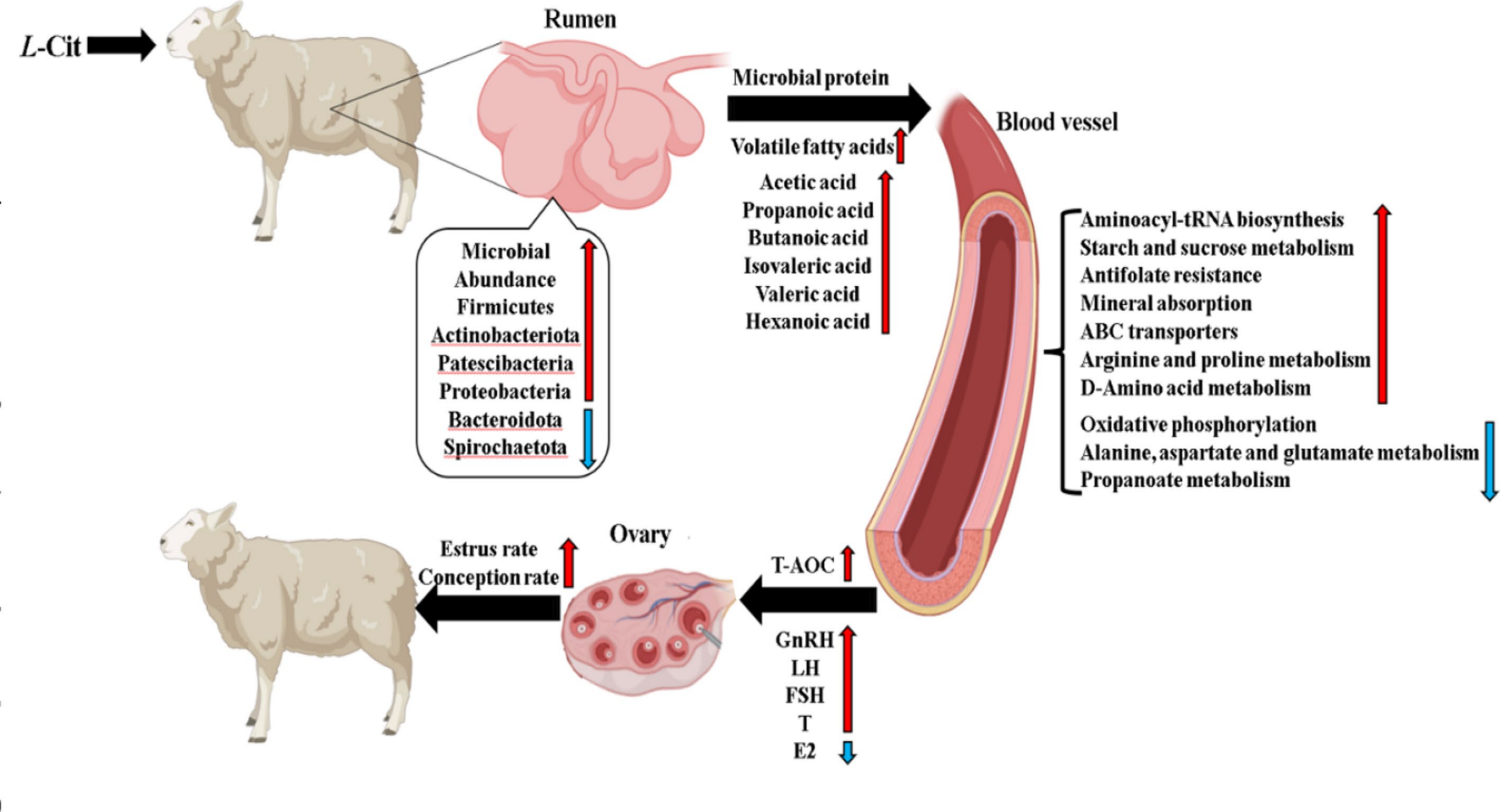
Synthesis figure of conclusions.

In this study, while no significant differences in plasma NO levels were observed, it is hypothesized that *L*-Cit supplementation may cross the pro-oxidative threshold of NO concentration in the local microenvironment, as suggested by the dose-effect characteristics of iNOS activation. This could explain the observed increase in MDA levels. A significant negative correlation was found between MDA content and the abundance of Bacteroidetes (*p* ≤ 0.05). Specifically, compared to the CON group, the relative abundance of Bacteroidetes was decreased in the experimental group, with the abundance of Prevotellaceae_UCG-003, a genus under Bacteroidetes, significantly downregulated (*p* = 0.04). Furthermore, a significant negative correlation was observed with MDA content (*p* ≤ 0.05), suggesting that specific Bacteroidetes microbiota may influence oxidative stress by modulating host metabolism. Further plasma metabolomic analysis revealed a significant negative correlation between MDA content and three downregulated metabolites: trans-4-aminocyclohexanecarboxylic acid, 2,8-quinolinediol-2-sulfate, and 2-methyl-6-{[(5-phenyl-2-thienyl)carbonylamino] benzoic acid} (*p* ≤ 0.05). These metabolites may indirectly influence lipid peroxidation through their involvement in antioxidant defense or signal transduction pathways (Figure 13).

## 6 Conclusion

Under the experimental conditions of this study, the following conclusions can be drawn: (Ma et al., 2019) Supplementation with L-citrulline was found to alter the rumen microbiota structure in Hu sheep ewes, resulting in an increased relative abundance of Firmicutes along with decreased relative abundances of Bacteroidetes and Proteobacteria.(Wu et al., 2018) *L*-Citrulline supplementation was demonstrated to significantly increase (*p* < 0.01) the ruminal concentrations of acetate, propionate, isovalerate, and caproate, while significantly elevating (*p* < 0.05) butyrate and valerate levels in Hu sheep ewes.(Cynober et al., 1995) L-citrulline supplementation was shown to enhance glucose metabolic efficiency and xenobiotic degradation capacity in the blood of Hu sheep ewes.(Curis et al., 2005) L-citrulline supplementation significantly improved reproductive hormone levels, estrus rate, and conception rate in Hu sheep ewes.

Therefore, the rumen microbiota structure in Hu sheep ewes is modified through supplemental feeding of L-citrulline, leading to an increase in ruminal volatile fatty acid content. This modification promotes the efficiency of blood glucose metabolism and the degradation capacity of exogenous substances in the organism, while relevant reproductive hormone levels are elevated. Consequently, both the estrus rate and conception rate are enhanced through these mechanisms.

## Data Availability

The datasets presented in this study can be found in online repositories. The names of the repository/repositories and accession number(s) can be found in the article/Supplementary material.
