# Supplementary material for: Effect of supplementation with L-Citrulline on rumen microbiota structure, plasma metabolites, reproductive hormones, and antioxidant capacity of Hu ewes
Source: Front Microbiol. 2025 Jun 25;16:1606437. doi: 10.3389/fmicb.2025.1606437 (PMC12243934; doi:10.3389/fmicb.2025.1606437)
Supplement: Supplementary file 1 [file Data_Sheet_1.docx]

**Table S1 Experimental design and grouping**

| Items | Quantity | TMR feeding | The feeding amount of *L*-citrulline |
| --- | --- | --- | --- |
| Control group   1. group | 30 | The daily TMR feeding amount for each sheep is approximately 1.65 kg/d (dry matter). | 0 g/d *L*-Cit |
| Experimental group (T) group | 30 |  | 10 g/d *L*-Cit |

**Table S2 Rumen pH Results**

| Item | Control group  (C) group | Experimental group  (T) group | *P*值  *P*-value |
| --- | --- | --- | --- |
| pH | 5.94±0.41 | 5.92±0.18 | 0.951 |

Note: Data in the table are presented as mean ± standard deviation.

**Table S3 The results of the alpha diversity analysis were obtained**

| Item | Control group  (C) group | Experimental group  (T) group | *P*-value |
| --- | --- | --- | --- |
| Sobs | 1362.83±189.14 | 1502.50±148.43 | 0.181 |
| Chao1 | 1365.58±191.25 | 1509.06±148.75 | 0.175 |
| Ace | 1372.18±196.25 | 1521.19±149.92 | 0.172 |
| Shannon | 6.27±0.29 | 6.26±0.34 | 0.971 |
| Coverage | 0.99±0.0005 | 0.99±0.0003 | 0.058 |
| Simpson | 0.005±0.003 | 0,007±0.008 | 0.410 |

Note: Data in the table are presented as mean ± standard deviation.

**Table S4 Relative Abundance of Volatile Fatty Acids**

| Item | Control group  (C) group | Experimental group  (T) group | *P*-value |
| --- | --- | --- | --- |
| Acetic acid | 67.58±16.31 | 118.98±33.67 | ＜0.01 |
| Propanoic acid | 3.49±0.77 | 7.31±1.63 | ＜0.01 |
| Butanoic acid | 2.31±1.39 | 4.59±1.48 | ＜0.05 |
| Isovaleric acid | 0.639±0.25 | 1.28±0.37 | ＜0.01 |
| Valeric acid | 0.15±0.04 | 0.21±0.03 | ＜0.05 |
| Hexanoic acid | 0.14±0.03 | 0.20±0.03 | ＜0.01 |

Note: Data in the table are presented as mean ± standard deviation.

**Table S5 Differential metabolites in positive ion mode**

| Metabolite | *P*_value | VIP | FC(T/C) | M/Z | Retention time/min | Adducts |
| --- | --- | --- | --- | --- | --- | --- |
| Dimethyl Sulfoxide | 0.01 | 3.40 | 1.21 | 79.02 | 0.81 | M+H |
| Carmofur | 0.00 | 3.62 | 1.19 | 258.12 | 2.77 | M+H |
| Flumazenil | 0.00 | 3.05 | 1.13 | 304.11 | 2.63 | M+H |
| Loxoribine | 0.01 | 2.85 | 1.12 | 322.12 | 5.40 | M+H-H2O |
| Subaphylline | 0.03 | 2.59 | 1.11 | 319.16 | 3.97 | M+CH3OH+Na |
| Biotin | 0.03 | 2.00 | 1.08 | 245.09 | 2.49 | M+H |
| Nitrotyrosine | 0.04 | 2.05 | 1.07 | 191.04 | 3.56 | M+H-2H2O |
| Asparaginylalanine | 0.03 | 1.99 | 1.07 | 186.09 | 0.80 | M+H-H2O |
| *L*-Prolinamide | 0.02 | 1.98 | 1.07 | 115.09 | 0.52 | M+H |
| Propionylcarnitine | 0.01 | 2.50 | 1.06 | 218.14 | 0.87 | M+H |
| 2-Isopropylmalic Acid | 0.01 | 1.82 | 1.05 | 159.06 | 1.93 | M+H-H2O |
| 8-Methoxykynurenate | 0.03 | 1.69 | 1.05 | 220.06 | 3.34 | M+H |
| Trimethylamine N-Oxide | 0.04 | 1.73 | 1.05 | 76.08 | 0.64 | M+H |
| *L-*Ornithine | 0.01 | 1.82 | 1.04 | 133.10 | 0.52 | M+H |
| 2-Furoylglycine | 0.02 | 1.61 | 1.04 | 170.04 | 2.78 | M+H |
| Ser Gln Ala | 0.03 | 1.63 | 1.04 | 305.14 | 0.79 | M+H |
| 2-Methylbutyroylcarnitine | 0.01 | 1.74 | 1.04 | 246.17 | 3.31 | M+H |
| *L*-Proline | 0.01 | 1.58 | 1.04 | 116.07 | 0.52 | M+H |
| Sucrose | 0.04 | 1.36 | 1.04 | 365.10 | 0.71 | M+Na |
| Tolmetin | 0.01 | 1.60 | 1.03 | 240.10 | 2.44 | M+H-H2O |
| 1-Pyrroline | 0.00 | 1.47 | 1.03 | 70.07 | 0.68 | M+H |
| 2(5H)-Furanone | 0.01 | 1.35 | 1.03 | 85.03 | 1.93 | M+H |
| 3-Oxindole | 0.04 | 1.32 | 1.03 | 134.06 | 3.59 | M+H |
| 3-Butadiene | 0.03 | 1.43 | 1.03 | 72.08 | 0.82 | M+NH4 |
| N(6)-Methyllysine | 0.01 | 1.43 | 1.02 | 193.15 | 0.63 | M+CH3OH+H |
| L-Methionine | 0.03 | 1.23 | 1.02 | 150.06 | 1.13 | M+H |
| N,N-Dimethylacetamide | 0.03 | 1.10 | 1.02 | 88.08 | 0.64 | M+H |
| Butirosina | 0.01 | 1.08 | 1.02 | 556.28 | 6.45 | M+H |
| *L*-Valine | 0.03 | 1.17 | 1.01 | 118.09 | 0.82 | M+H |
| Armillaramide | 0.01 | 1.13 | 0.99 | 555.52 | 6.13 | M+ |
| 1,4-Ipomeadiol | 0.01 | 1.07 | 0.99 | 171.10 | 6.62 | M+H |
| Metyrosine | 7.34E-05 | 1.20 | 0.99 | 196.10 | 1.93 | M+H |
| Aminopentol | 0.00 | 1.38 | 0.98 | 438.38 | 6.74 | M+CH3OH+H |
| 2,4-Quinolinediol | 0.00 | 1.46 | 0.98 | 162.05 | 3.71 | M+H |
| Furfural | 0.02 | 1.20 | 0.98 | 97.03 | 0.74 | M+H |
| N-Dodecylsarcosinate | 0.02 | 1.41 | 0.98 | 290.27 | 6.15 | M+CH3OH+H |
| Indole-3-Carboxylic Acid | 0.01 | 1.33 | 0.98 | 162.05 | 4.44 | M+H |
| 1-Methyladenosine | 0.01 | 1.26 | 0.98 | 282.12 | 0.83 | M+H |
| N-Acetyl-L-Alanine | 0.04 | 1.35 | 0.97 | 132.07 | 0.74 | M+H |
| Cyclopentanol | 0.01 | 1.63 | 0.97 | 104.11 | 0.61 | M+NH4 |
| 1,6-Hexanediamine | 0.00 | 1.83 | 0.96 | 117.14 | 7.71 | M+H |
| 8-Hydroxyquinoline | 0.00 | 2.22 | 0.95 | 146.06 | 3.58 | M+H |
| Turicine | 0.01 | 1.97 | 0.94 | 160.10 | 0.83 | M+H |
| F1Goshonoside F1 | 0.00 | 2.65 | 0.91 | 484.30 | 6.43 | M+ |
| Tranexamic Acid | 0.01 | 2.69 | 0.89 | 180.10 | 0.85 | M+Na |
| *L*-Theanine | 0.02 | 2.96 | 0.87 | 157.10 | 2.10 | M+H-H2O |
| N-Acetylasparagine | 0.00 | 3.55 | 0.83 | 175.07 | 0.79 | M+H |
| N-Acetylhistidine | 0.02 | 3.64 | 0.75 | 215.11 | 0.78 | M+NH4 |
| Salicyluric Acid | 0.02 | 4.15 | 0.72 | 250.07 | 4.88 | M+CH3OH+Na |
| *L*-Norleucine | 0.00 | 4.47 | 0.72 | 173.13 | 0.67 | M+ACN+H |

**Table S6 Differential metabolites in negative ion mode**

| Metabolite | *P*_value | VIP | FC(T/C) | M/Z | Retention time/min | Adducts |
| --- | --- | --- | --- | --- | --- | --- |
| Nervonic Acid | 0.05 | 1.30 | 1.03 | 411.35 | 6.93 | M+FA-H |
| Coixinden B | 0.02 | 1.61 | 1.03 | 215.07 | 1.70 | M-H2O-H |
| Isonicotinic Acid | 0.05 | 1.26 | 1.03 | 168.03 | 3.25 | M+FA-H |
| Indolelactic Acid | 0.01 | 1.27 | 1.02 | 204.07 | 5.52 | M-H |
| Methylmalonic Acid | 0.01 | 1.40 | 0.98 | 117.02 | 1.55 | M-H |
| Succinic Acid | 0.02 | 1.36 | 0.97 | 117.02 | 1.05 | M-H |
| 1,17-Heptadecanediol | 0.04 | 1.50 | 0.96 | 293.25 | 6.51 | M+Na-2H |
| 7-Methylrosmanol | 0.04 | 1.70 | 0.95 | 359.19 | 6.01 | M-H |
| Arabinogalactose | 0.03 | 1.68 | 0.95 | 357.10 | 0.83 | M+FA-H |
| Inosine | 0.04 | 1.88 | 0.93 | 267.07 | 1.92 | M-H |
| N-Formylmethionine | 0.00 | 2.53 | 0.90 | 176.04 | 3.61 | M-H |
| 7-Amino-4-Methylcoumarin | 0.01 | 3.16 | 0.82 | 210.03 | 5.93 | M+Cl |
| *L*-Histidinol | 0.00 | 4.66 | 0.58 | 186.09 | 0.78 | M+FA-H |


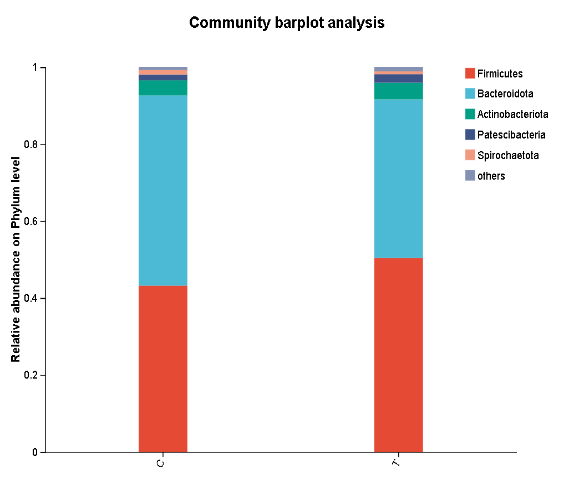


A


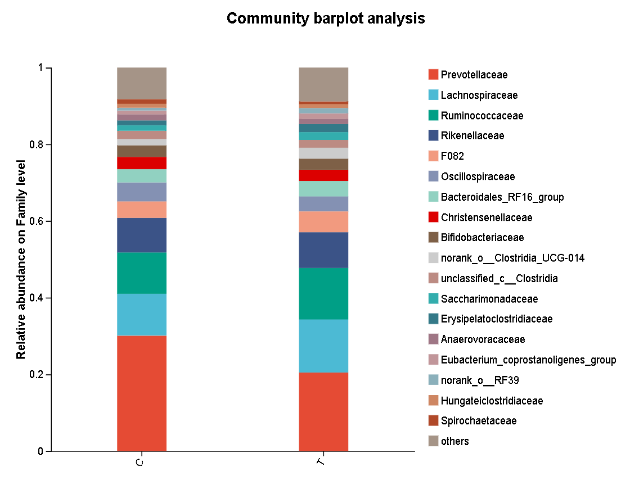


B


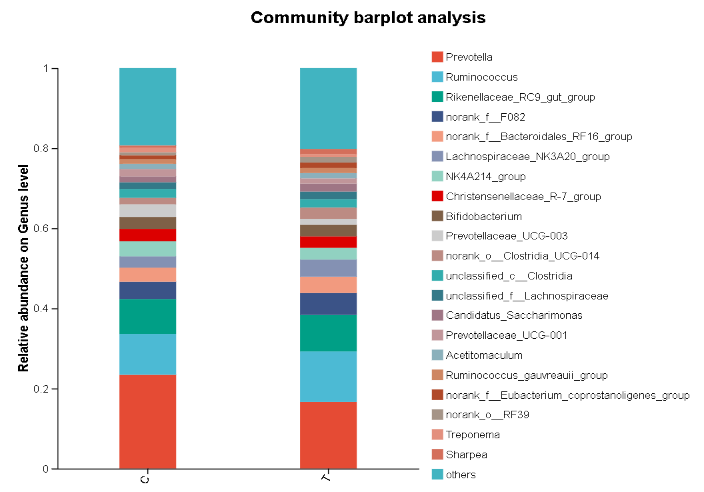


C

**Fig S1 The relative abundance of rumen microbiota was analyzed and visualized at different taxonomic levels**

**A: Phylum level, B:Family level, C: Genus level**

Note: C denotes the control group; T represents the experimental group.
